# Systematic Evaluation of Depletion and Enrichment Technologies for Platelet-Free Plasma Proteomics

**DOI:** 10.1021/acs.jproteome.5c01056

**Published:** 2026-03-17

**Authors:** Salem Al Siblani, Jean Armengaud, Clément Lozano

**Affiliations:** † Institut Hospitalo-Universitaire Comprehensive SEPSIS Center, Paris-Saclay University, 91190 Saclay, France; ‡ Département Médicaments et Technologies pour la Santé (DMTS), 84250 Université Paris-Saclay, CEA, INRAE, SPI, 30200 Bagnols-sur-Ceze, ` France; Laboratoire Innovations Technologiques pour la Détection et le Diagnostic (Li2D), Université de Montpellier, F-30207 Bagnols-sur-Ceze, France; § Université Paris-Saclay, CEA, INRAE, Département Médicaments et Technologies pour la Santé (DMTS), SPI, 30200 Bagnols-sur-Cèze, France

**Keywords:** plasma proteomics, platelet-free plasma, PCA-N, immunodepletion, ProteoMiner, MagNet-SAX, ENRICHplus, Proteonano, ASTRAL, DIA

## Abstract

Plasma proteomics is a rapid, noninvasive, and highly informative approach for identifying disease biomarkers. However, the wide dynamic range of protein concentration limits the depth of liquid chromatography–tandem mass spectrometry proteomics. To address this challenge, we evaluated, with an Orbitrap Astral instrument using DIA, the performance of several protein depleting and enriching technologies on platelet-free plasma. Specifically, we assessed: perchloric acid depletion, immunodepletion, ProteoMiner, MagNet-SAX, ENRICHplus, Proteonano and the combination of ProteoMiner and immunodepletion. All methods were assessed in terms of proteomic depths, protein quantification precision, and functional analysis. Proteonano exhibited the most effective enrichment for the lowest abundance proteins, confidently identifying 299 proteins with mapped blood concentrations below 10^6^ pg/L. Immunodepletion yielded the highest proteome coverage in the moderate abundance range (660 confident proteins). Also, ENRICHplus quantitative profile closely matched that of the neat plasma (93% correlation). Additionally, high repeatability (median coefficient of variation) was demonstrated by MagNet-SAX (13%), ProteoMiner (15%), and immunodepletion (16%). Combining ProteoMiner and immunodepletion reduced the plasma protein dynamic range, enabling deeper low abundance protein analysis but decreased repeatability. These results obtained on platelet-free plasma deviate from previously reported results on platelet-rich plasma, highlighting the crucial sample preparation stage for plasma proteomics.

## Graphical abstract

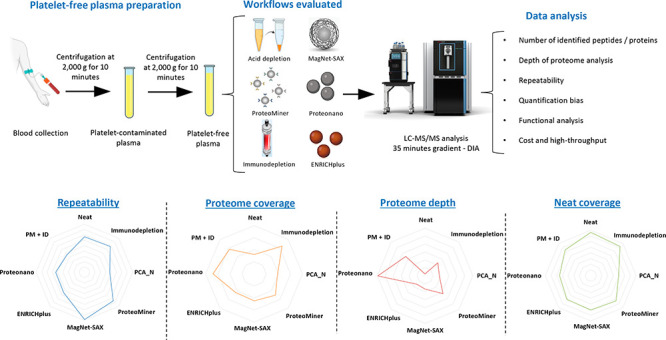

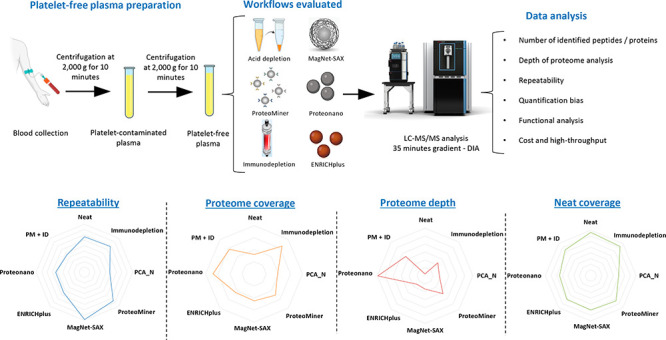

## Introduction

Human blood plasma is a valuable source for biomarker discovery because it can be collected with minimal invasiveness, is readily accessible, and reflects physiological and pathological states across multiple organs.^1^ However, plasma harbors one of the most complex proteomes among all biofluids, encompassing thousands of proteins secreted from tissues, immune cells, and circulating blood components.^2^ Its extremely wide dynamic range, with protein concentrations spanning over 12 orders of magnitude, is one of the major analytical challenges since analyzers from state-of-the-art mass spectrometers display an intrascan dynamic range of 1000.^3^ The plasma proteome is dominated by some 20 highly abundant proteins including albumin, immunoglobulins, transferrin, and haptoglobin, which account for approximately 99% of the total protein content, and hamper the detection and quantification of lower abundant proteins, many of which may hold diagnostic or prognostic value.^3^

To improve the proteome coverage when analyzing plasma samples, various protein depletion, equalization, and enrichment strategies have been developed.^4^ Classical chemical precipitation techniques such as perchloric acid (PercA),^5^ methanol (MeOH),^6^ trichloroacetic acid (TCA),^6^ and polyethylene glycol (PEG)^7^ have been employed to deplete highly abundant proteins that tend to precipitate faster than the lower abundant proteins. Perchloric acid precipitation with a neutralization step (PCA_N) has recently been described as an improvement over the conventional PercA approach in which the depleted sample is neutralized following acid treatment. This enables direct protein digestion of the neutralized sample, thereby increasing throughput and minimizing sample loss.^8^ Other approaches rely on immune affinity-based depletion of abundant proteins (e.g., Pierce Top 14 (ThermoFisher), Multiple Affinity Removal System MARS (Agilent), and Seppro IgY14 spin columns (Sigma-Aldrich)) by utilizing antibodies to selectively retain the top abundant proteins, leading to increased identification of low-abundant proteins. In order to further increase the proteome coverage, combinatorial peptide ligand libraries have been proposed. For example, the ProteoMiner (Bio-Rad)^9^ relies on the protein equalization concept, where proteins bind to a diverse library of hexapeptides with varying affinities, but equal quantities of each protein are retained. This binding process leads to the depletion of high abundance proteins, which saturate their specific ligands, and the enrichment of low abundance proteins that have available binding sites on other hexapeptides. More recently, functionalized nanoparticles have been developed, adopting the same strategy for capturing low-abundant proteins. Several kits are available such as ENRICHplus (PreOmics) magnetic beads for the enrichment of low-abundant proteins. MagNet-SAX (ReSyn Biosciences) comprises superparamagnetic polymer microparticles with surface modification by quaternary ammonium groups, which confer strong anion-exchange (SAX) properties, the latter preferably targeting the content of extracellular vesicles (EVs).^10^ A recently introduced solution, Proteonano (Nanomics) utilizes peptide-conjugated nanoparticles for the enrichment of low-abundant proteins.^11^ Finally, Proteograph (Seer)^12^ relies on the protein corona formation around diverse surface chemistry nanoparticles for the enrichment of low-abundant proteins. Once low abundance proteins are enriched, proteins are identified and quantified after trypsin proteolysis and high-resolution tandem mass spectrometry of the resulting peptides.

Sample processing is a critical preanalytical factor in plasma proteomics. Collection and centrifugation protocols to prepare the plasma strongly influence the resulting proteome.^13^ In particular, platelets represent a major effector. Although platelet proteins can provide insights into cardiovascular, coagulation, and inflammatory states,^14–17^ they are prone to ex vivo activation during plasma preparation, releasing proteins and EVs that introduce biases in biomarker studies.^13,18^ Indeed, approximately 99% of EVs in circulation are hematopoietic in origin, with ∼50% arising from platelets.^19^ This complicates the interpretation of EV-associated proteomics, particularly when discriminating platelet-derived proteins from disease-relevant cell-free or tissue-derived biomarkers.^20^ The presence of platelet proteins has affected the performance of various protein depletion and enrichment techniques, introducing a contamination-induced boost in the identified proteome.^13^ To limit platelet contamination, double centrifugation of plasma should be applied (2000 g for 10 min), resulting in what is referred to as platelet-free plasma.^20,21^ However, the terminology is debated, as trace platelet-derived components remain even in platelet-free plasma.^22^ These preanalytical steps have increasingly been recognized as critical to ensuring reproducibility, sensitivity, and low-bias quantification in plasma proteomic studies, particularly in the context of biomarker discovery using both LC–MS/MS and antibody/aptamer-based platforms.^13^

Several studies have compared different plasma proteomics approaches, focusing mainly on the number of proteins identified, reproducibility, and functional analysis^5,10,23–26^ with limited consideration of the protein quantification bias. Additionally, most of these evaluations have relied on plasma prepared using low-speed centrifugation, i.e., platelet-contaminated plasma. While many recent studies have evaluated and benchmarked plasma proteomics workflows, differences in plasma preparation protocols can lead to variability even among samples described as platelet-free plasma.^21^ For examples, Beimers et al.^23^ benchmarked multiple plasma proteomics technologies using single-step centrifugation (3000 × g for 5 min, EDTA); Kircher et al.^26^ used plasmapheresis with sodium citrate; Roger et al.^27^ applied double centrifugation at 1500 × g for 10 min; Star et al.^28^ combined 1500 × g and 10,000 × g spins for extracellular vesicle isolation benchmarking; Gao et al.^29^ evaluated nanoparticle-based workflows after double centrifugation at 2000 × g for 15 min; and finally, Järvinen et al.^30^ benchmarked automated Mag-Net enrichment workflows after 1500 × g centrifugation. These studies provide highly valuable workflow comparisons, including the assessment of contamination markers. However, differences in plasma preparation, particularly the centrifugation speed and duration, can produce divergent results. Thus, we aimed to systematically benchmark seven workflows using plasma prepared by two consecutive centrifugation steps (10 min at 2000 × g) to enable evaluation under highly platelet-depleted conditions. Recently, more interest have been directed toward the protein quantification bias.^13,27,31^ In this study, we compared the protein quantification performance of several approaches for reducing the dynamic range of plasma proteins, including perchloric acid precipitation (PCA_N), MagNet-SAX, immunodepletion, ENRICHplus (prereleased kit), ProteoMiner, and Proteonano. The benchmarking focused on several aspects of plasma proteomics, particularly the landscape of peptides and proteins identified. We also evaluated the proportion of confidently identified proteins using each workflow. To assess coverage across the proteome dynamic range, the identified proteins were mapped to the reported blood concentration in the Human Protein Atlas. In addition, functional analysis of the identified proteome by the various technologies was conducted using specific markers to discriminate platelet proteins from EVs. Finally, the evaluation addressed workflow-induced protein quantification bias across technical replicates, providing insights into protein quantification enrichment and precision.

## Materials and Methods

### Platelet-Free Plasma Preparation

Peripheral blood was collected from a healthy adult donor in a 10 mL EDTA tube (reference 367525; 18 mg K2EDTA, BD Vacutainer K2E) and gently inverted to ensure anticoagulant mixing. Within 60 min of collection, it was centrifuged at 2000*g* for 10 min at 4 °C. The plasma supernatant was carefully aspirated without disturbing the buffy coat. The supernatant was further centrifuged at 2000*g* for 10 min at 4 °C. The resulting supernatant was aspirated without the bottom 200 μL. The resulting platelet-free plasma was then aliquoted and stored at −20 °C until further processing.

### Perchloric Acid with Neutralization

Platelet-free plasma (5 μL) was diluted in 20 μL of Milli-Q H2O, followed by the addition of 25 μL of 10% perchloric acid, obtained from the dilution of 60% perchloric acid (311413, Sigma-Aldrich) in Milli-Q water. Samples were agitated at 4 °C for 1 h, followed by centrifugation at 4000×g for 20 min at 4 °C. The supernatant (24 μL) was collected and neutralized by the addition of 8 μL 1.4 M sodium hydroxide solution.^8^ The depleted fraction was digested by SP3 digestion and desalted with C18, as described later. This condition is referred to as PCA_N.

### ProteoMiner

ProteoMiner (reference 163-3006, Bio-Rad) was used according to the manufacturer’s protocol. Briefly, the top and bottom caps were removed from spin columns; the columns were placed in the collection tube and centrifuged at 1000*g* for 1 min to remove the storage solution. The columns were washed with 200 μL of wash buffer and rotated on a roller mixer (SRT6, Stuart) for 5 min, followed by centrifugation at 1000*g* for 1 min. Washing was repeated three times. Then, 200 μL of platelet-free plasma was added to the column, which was then rotated on a roller mixer for 2 h. After binding, the bottom cap was removed, and the column was centrifuged in a collection tube to remove unbound material. Then, columns were washed three times with 200 μL of wash buffer, each step including rotation on a roller mixer for 5 min and centrifugation at 1000*g* for 1 min. After all wash buffer was removed, 200 μL of Milli-Q water was added to the column, rotated on a roller mixer for 1 min, placed in a collection tube, and centrifuged at 1000*g* for 1 min. Then, 20 μL of rehydrated elution reagent was incubated for 15 min with gentle vortexing every 5 min. The proteins were then eluted by placing the column in a collection tube and centrifuging at 1000*g* for 1 min. The elution process was repeated for a total of three times. The ProteoMiner equalized protein fraction was digested with SP3 digestion and desalted with C18, as mentioned below.

### Immunodepletion

Depletion spin columns (ref A36369, High Select 14, Thermo Fisher Scientific) were equilibrated to room temperature prior to use. A volume of 10 μL of platelet-free plasma was applied directly to the resin slurry within the column. The column was recapped and inverted several times until the resin was thoroughly resuspended and homogeneous. The mixture was incubated for 10 min at room temperature on a roller mixer to facilitate interaction between the sample and the resin. Following incubation, the bottom closure was removed, and the top cap was loosened. The column was placed into a 2 mL collection tube and centrifuged at 1000*g* for 2 min. The flow-through material was collected, and the resin-containing column was discarded. The immunodepleted flow-through was digested with SP3 digestion and desalted with C18, as mentioned below.

### ProteoMiner Followed by Immunodepletion—PM + ID

The sequential combination of ProteoMiner and immunodepletion processing of the platelet-free plasma was performed using the two previously mentioned protocols. First, 200 μL of the platelet-free plasma was processed using the ProteoMiner kit. The proteins eluted from the ProteoMiner column were directly placed in the immunodepletion column and processed as described above. The flow-through was collected (∼350 μL), and the resin-containing column was discarded. To concentrate the proteins in the flow-through and enable consistent input volumes for downstream processing, proteins were desalted using HLB columns (AttractSPE Disks Spin HLB, Spin-HLB.T1.96, Affinisep) as previously reported.^5^ This step decreased the sample volume to 50 μL, allowing the complete protein content to be processed in a single SP3 digestion reaction, as described in the SP3 section below. Following proteolysis, peptides were desalted using C18 columns, as described for all other workflows. The samples were then dried using Speedvac at 50 °C and then resuspended in 50 μL of PBS1X (Thermo Fisher Scientific). The concentrated proteins, treated with PM + ID, were digested with SP3 digestion and desalted with C18, as described below.

### MagNet-SAX

The MagNet-SAX method was performed based on the manufacturer’s protocol. All steps were performed in a 96-well plate format using LoBind 0.5 mL plates (951032107, Eppendorf). Briefly, 12.5 μL of MagReSyn-SAX beads (reference MR-SAX005, ReSyn) were equilibrated twice in 200 μL of BTP equilibration/wash buffer (50 mM bis-tris propane pH 6.4 (Sigma-Aldrich) and 150 mM sodium chloride (Sigma-Aldrich)). After 30 s, the beads were held with a magnetic rack (MAGJET RACK, Thermo Fisher Scientific), and the supernatant was discarded. 50 μL of bind buffer (100 mM bis-tris propane pH 6.4, 150 mM sodium chloride) and 50 μL of platelet-free plasma were added to the beads and mixed at 450 rpm for 30 min on a Thermomixer. The magnetic beads were held with a magnetic rack, and the supernatant was discarded. For washing, 500 μL of BTP equilibration/wash buffer was added to each sample and mixed for 5 min at 450 rpm, and then beads were held with a magnetic rack while the supernatant was discarded. The washing was repeated a total of 3 times. Beads were then resuspended in 100 μL of lysis and reduction mix (50 mM Tris pH 8 (Sigma-Aldrich), 1% (w/v), sodium dodecyl sulfate (SDS) (Sigma-Aldrich), 10 mM DL-Dithiothreitol (DTT) (Sigma-Aldrich)) and incubated at 37 °C for 60 min while mixing at 450 rpm. Then, iodoacetamide (IAA) (Sigma-Aldrich) was added to a final concentration of 15 mM and incubated for 30 min in the dark. To induce on-bead precipitation, acetonitrile was added to a final concentration of 70% to each well, resuspended up and down three times, and incubated at RT for 10 min. The plate was moved to a magnetic rack, and the supernatant was removed. With the plate on the rack, 95% acetonitrile was added, and the mixture was incubated for 30 s and then removed. Washing was repeated three times. For on-bead digestion, 200 μL of digestion solution composed of 1 μg of trypsin gold in 25 mM ammonium bicarbonate was added and incubated at 47 °C for 2 h while mixing at 450 rpm. Digestion was quenched with trifluoroacetic acid (Fisher Chemical) to a final concentration of 0.5%. Peptides were desalted on a C18 solid-phase extraction cartridge. The peptides were then dried using Speedvac at 50 °C. Dried peptides were resuspended in 50 μL of Milli-Q water, and peptides were quantified by fluorometric quantification (Pierce quantitative peptide assays, ref 23930, Thermo Fisher Scientific) and then acidified to a final concentration of 0.5% trifluoroacetic acid.

### Nanomics Proteonano

Protein enrichment was achieved utilizing Proteonano Plasma Proteome Enrich kit (Nanomics) according to manufacturer’s instructions. Briefly, 40 μL of EN-Binding buffer was placed in a LoBind 0.5 mL plate, and 40 μL of platelet-free plasma was added. The plate was then mixed using a Thermomixer at RT for 5 min at 450 rpm. Then, 20 μL of Enrichment Nanobeads were added to each sample, beads were resuspended gently up and down using a pipet for a total of three times, and the plate was mixed at RT on a Thermomixer for 1 h at 450 rpm. After incubation, the plate was placed on a magnetic rack for 3 min and the unbound supernatant was discarded. The sample was then washed by adding 180 μL of EN-Wash Buffer, then resuspended gently up and down using a pipet for a total of three times. The plate was then placed on a magnetic rack for 3 min (until the beads are totally retained), and the supernatant was discarded. This washing step was repeated three times. The proteins were reduced and alkylated by adding 4 μL of a 35 mM DTT solution and 4 μL of a 105 mM IAA solution and incubating for 30 min at 55 °C in the dark. For digestion, 1 mL of Digestion Buffer 2 was transferred to the Enzyme bottle, vortexed thoroughly, and 20 μL of this solution was added to each sample. The plate was incubated at 37 °C by using a Thermomixer at 450 rpm for 2 h in the dark. To stop digestion, 20 μL of the ENDING Buffer was added and mixed for 3 min using a Thermomixer at RT at 450 rpm. The plate was then placed on a magnetic rack until the beads were totally retained and the supernatant was collected. Peptides were desalted on C18 solid-phase extraction cartridge. The peptides were then dried using Speedvac at 50 °C. Dried peptides were resuspended in 50 μL of Milli-Q water, quantified by fluorometric quantification, and acidified to a final concentration of 0.5% trifluoroacetic acid.

### PreOmics ENRICHplus

Protein enrichment was performed using the prereleased ENRICHplus kit (PreOmics) according to manufacturer’s protocol. All steps were performed in a 96-well plate format using a LoBind 0.5 mL plate. First, 200 μL of ENplus-WASH was added to each well and mixed with 25 μL of ENplus-BEADS (beads were thoroughly resuspended before addition to wells). The plate was then mixed at 450 rpm for 1 min using a Thermomixer. Beads were retained using a magnetic rack, and the supernatant was discarded. Washing was repeated three times. Volumes of 50 μL of platelet-free plasma and 50 μL of ENplus-BIND buffer were added to the beads and mixed for 30 min at 450 rpm and 30 °C. The plate was then placed on a magnetic rack, and the supernatant was discarded. For the washing, 100 μL of ENplus-BIND was added and mixed at 450 rpm for 1 min. Then, the plate was placed on the magnetic rack and the supernatant was discarded. Washing was repeated again three times. LYSE and BIND buffers were mixed, and 40 μL was added to each well and mixed for 10 min at 450 rpm and 60 °C. The samples were cooled down to RT. Then, 525 μL of RESUSPEND was added to the DIGEST, and mixed thoroughly. A volume of 10 μL of DIGEST was then added to each sample and mixed at 450 rpm and 37 °C for 1 h. Digestion was stopped by adding 100 μL of the STOP solution. Peptides were desalted on a C18 solid-phase extraction cartridge. The peptides were then dried using Speedvac at 50 °C. Dried peptides were resuspended in 50 μL of Milli-Q water, and peptides were quantified by fluorometric quantification and then acidified to a final concentration of 0.5% trifluoroacetic acid.

### SP3 Digestion

The Speedbeads magnetic carboxylate-modified particles (GE45152105050250 and GE65152105050250, GE Healthcare) were combined in a 1:1 (v/v) ratio. The beads were washed twice with Milli-Q water. The beads were resuspended in Milli-Q water to obtain final concentrations of 50 mg/mL. Volumes of 50 μL of the proteins obtained from the processing of PCA_N (24 μL of PCA_N processed proteins with 26 μL of PBS1X), PM+ID (50 μL of the processed proteins), immunodepletion (50 μL of the processed proteins), ProteoMiner (50 μL of the processed proteins), and the neat (1 μL platelet-free plasma with 49 μL of PBS1X) were placed in the LoBinding 96-well plate. The proteins were reduced and alkylated by adding 4 μL of 35 mM DTT solution and 4 μL of 105 mM IAA solution and mixing for 10 min at room temperature in the dark at 450 rpm using a thermomixer.

SP3 protocol was conducted as previously described^32^ with slight changes. Briefly, 4 μL of magnetic beads were added to the reduced and alkylated samples. Acetonitrile was then added to reach a final concentration of 85% followed by incubation at room temperature for 2 min. The beads were placed on a magnetic rack, and the supernatant was discarded. Samples were then washed twice by adding 200 μL of 70% ethanol and once with 180 μL of acetonitrile. The 96-well plate was left under the hood with the plate cover on for 2 min to evaporate any remaining acetonitrile. On-bead proteolysis was achieved by adding 30 μL of digestion solution composed of 0.1 μg of trypsin gold (Promega) in 50 mM ammonium bicarbonate and incubating at 50 °C for 1 h. Peptides were desalted on a C18 solid-phase extraction cartridge. The samples were then dried using a Speedvac at 50 °C. Dried peptides were resuspended in 50 μL of Milli-Q water, and peptides were quantified by fluorometric quantification and then acidified to a final concentration of 0.5% trifluoroacetic acid.

### C18 Desalting

Peptides were desalted using C18 spin columns (AttractSPE Disks Spin C18, Spin-C18.T1.96, Affinisep) according to the manufacturer’s recommendations with minor modifications. Columns were preconditioned by two sequential washes with 50 μL of acetonitrile (ACN)/0.1% trifluoroacetic acid (TFA) (99.9:0.1, v/v), followed by a wash with 50 μL of ACN/H_2_O/0.1% TFA (80:19.9:0.1, v/v/v) and equilibration with 50 μL of ACN/H_2_O/0.1% TFA (2.5:97.4:0.1, v/v/v). Columns were subsequently equilibrated with 200 μL of 0.5% acetic acid in water. Samples were then loaded onto the columns and washed with 200 μL of 0.5% acetic acid. Peptides were eluted in two sequential steps using 50 μL of H_2_O/ACN (20:80, v/v). All centrifugation steps were performed at 500×*g* for 1.5 min at room temperature. The same desalting protocol was used for all workflows.

### Liquid Chromatography—Tandem Mass Spectrometry

Acidified peptides (150 ng) were injected per sample and analyzed with an Orbitrap Astral mass spectrometer (Thermo Electron) coupled to a Vanquish Neo UHPLC system (Thermo Electron). Peptides were desalted on a reversed-phase PepMap 100 C18 trapping column (5 μm, 300 μm × 5 mm) and resolved on a 25 cm Aurora Ultimate column (25 cm × 75 μm ID, 1.7 μm C18, IonOpticks) at a flow rate of 0.4 μL/min. The separation was performed using a 35 min gradient (3–25% B from 0 to 30 min, 25–36% B from 30 to 35 min) of mobile phase A (0.1% HCOOH/99.9% H2O) and phase B (0.1% HCOOH/99.9% CH_3_CN). MS1 was acquired in Orbitrap mode with 240,000 resolution every 0.6 s. The MS1 normalized AGC target was set to 500% (5 × 10^6^ charges) with a 10 ms maximum injection time of 40% RF lens. The mass spectrometer was operated in data-independent acquisition mode (DIA) with precursor ion selection for fragmentation ranging between *m*/*z* 380 and 980, using 300 isolation windows of *m*/*z* 2 with no overlap between adjacent windows. For the MS2, the normalized AGC target was set to 500% (5 × 10^6^ charges) with a 3 ms maximum injection time.

### Data Interpretation and Statistical Analysis

Following LC–MS/MS acquisition, raw spectra files were interpreted using DIA-NN^33^ (2.2.0) and the UniProtKB/Swiss-Prot Human database (released in 2025_04; 20663 protein sequences). The following settings were applied: FASTA digest for library-free search/library generation, deep learning-based spectra, RTs and IMs prediction were selected; maximum number of miscleavages = 2; maximum number of variable modifications = 1, modifications = N-term M excision, C carbamidomethylation, M oxidation; Match between runs (MBR) was not selected; precursor false discovery rate = 0.01. The output of DIA-NN was processed using R (version 4.5.1), RStudio (version 2024.12.1), and Excel version 2007. Protein groups were considered identified when they met the following DIA-NN filtering criteria: Q.Value ≤ 0.01, Global.Q.Value ≤ 0.01, PG.Q.Value ≤ 0.05, and Global.PG.Q.Value ≤ 0.01.

One-way ANOVA was used to assess differences in protein numbers between experimental conditions, followed by Tukey’s Most Significant Difference (HSD) test for pairwise comparisons (*P* < 0.05). The majority of the plots were created using ggplot2 (version 4.0.0) embedded in tidyverse.^34^ Upsetplot was created using UpSetR (version 1.4.0) package^35^ in R. Protein abundance stacked bar charts were produced using ggplot2 after data processing with readxl (version 1.4.5), dplyr (version 1.1.4), tidyr (version 1.3.1), and writexl (version 1.5.4). Significance between conditions was assessed using Wilcoxon rank-sum tests, with multiple testing correction performed using the Benjamini-Hochberg procedure to calculate adjusted *P*-values. Distributions were visualized using box plots and jitter overlays, and significant differences (*P* < 0.05) were exported to Excel. Color palettes were provided by Viridis (version 0.6.5) package in R. For improved visuals, labels of the graphs were modified using Stringr (version 1.5.2) package in R. GGally (version 2.4.0) package in R was used for the pairwise correlation scatterplot. Gene Ontology (GO) functional analysis was performed using g:Profiler^36^ using *Homo sapiens* database with a gSCS threshold of 0.05, and the full identified list of proteins was used as a custom background. For particular functional analysis and protein abundance concentration in blood, Human Protein Atlas (Human Protein Atlas proteinatlas.org)^37^ was used. The evaluation of the platelet contamination index was based on Baize software according to the intensity of specific platelet markers (https://www.guomics.com/software/Baize). Venn diagrams were generated using InteractiVenn.^38^

For the evaluation of protein quantification bias, the log2-transformed LFQ intensity of each protein was extracted from three technical replicates per condition. Only proteins consistently detected in all replicates of both the reference (Neat plasma) and the tested conditions were used. For each protein, the mean log2 intensity across the Neat replicates was used as a reference. Fold changes (FC) per replicate were calculated as the difference between the log2 intensity of the replicate under the tested conditions and the Neat average (log2FC = log2­[Condition] – log2­[Neat_avg]). The average log2FC across replicates was used as the condition-specific fold change value (log2FC_Mean), denoted as “FC,” and presented in a heatmap. The variability of this measurement (log2FC_SD), denoted as “FC_SD,” was quantified as the standard deviation of replicate-specific log2FC values. For visualization, FC values were displayed as heatmaps using the ggplot2 package in R, and FC-SD values were grouped into windows to assess precision across conditions.

### Ethical Statement

The use of human samples in this study was authorized under the CODECOH agreement (agreement number: AC-2020-3959), in compliance with national and institutional ethical regulations, under the supervision of the Ministry of Education and Research. Donors provided informed consent for the use of their samples for research purposes. Samples were processed in an anonymized manner, and no identifiable information was accessible to the investigators.

## Results

### Strategy for Comparing Methods to Extend the Proteomic Coverage of Plasma

The study focuses on the evaluation of methods aimed at broadening proteome coverage of platelet-free plasma. A blood sample from a healthy individual was treated to obtain plasma and remove platelets through two consecutive centrifugation steps. Baize software evaluates the platelet, red blood cells and coagulation contamination indices of a sample based on the relative intensity of 30, 31, and 20 marker proteins, respectively.^29^ These contamination marker proteins were deduced from controlled platelet, erythrocyte, and coagulation spike-in experiments by identifying proteins whose abundances strongly and reproducibly increased with contamination levels, distinguishing them from platelet-independent circulating plasma proteins. The results of the interpretation with Baize software confirmed the low platelet (Figure 1) and red blood cell contents in the platelet-free plasma used in our study (Figure S1, panel A). The blood coagulation index reached a value of 10, exceeding the contamination threshold defined by the software (threshold = 1) (Figure S1, panel B). However, this threshold may not be optimal, as it does not align with the contamination indices reported in the reference study, where the majority of samples exhibited values of above 20. The platelet-low content of the sample was further confirmed by the absence of platelet-relevant markers that are not included within the Baize algorithm: CD41, CD42a, and CD61, while CD62p was quantified in low traces (Table S1).

**Figure 1:**
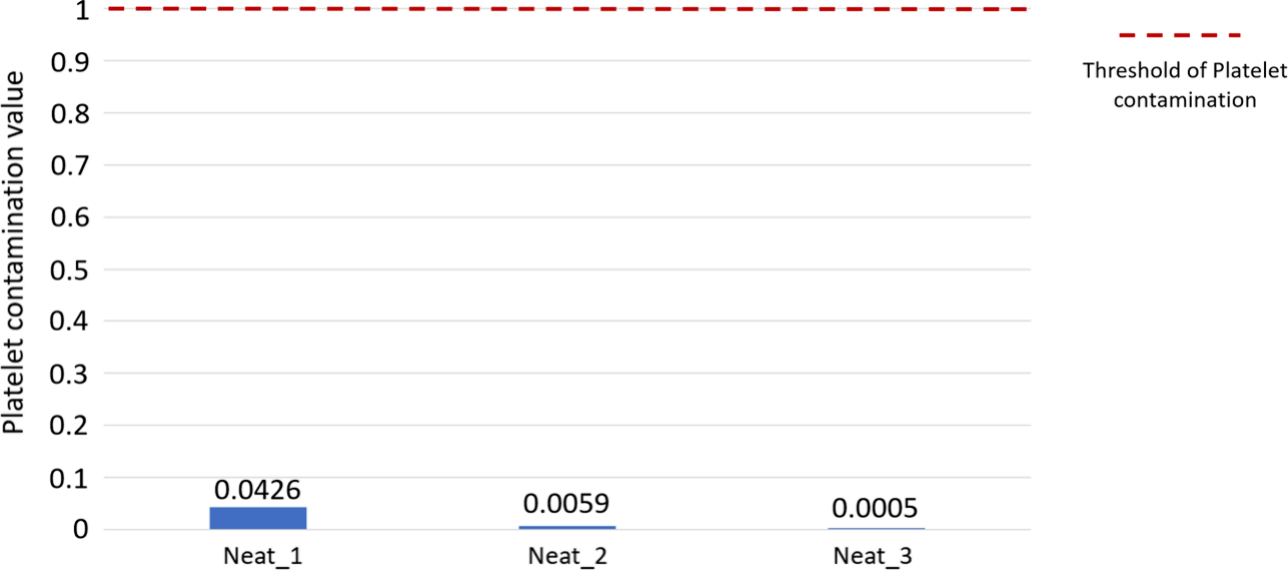
Platelet contamination values in neat plasma were estimated with Baize software. Values were log10-transformed, shifted to positive, and normalized so that the contamination threshold is equal to 1 (*y*plot = log_10_(*y*) – min­[log_10_(*y*)] + ϵ).

As depicted in Figure 2, to increase the proteome coverage of the platelet-depleted plasma, six enrichment/depletion workflows were evaluated along with the neat plasma, each performed in three technical preparation replicates: PCA_N, immunodepletion, ProteoMiner, MagNet-SAX, ENRICHplus, Proteonano, and the sequential workflow of ProteoMiner followed by Immunodepletion (PM+ID). Each workflow was performed in three independent sample preparation replicates to assess the technical variability introduced during the workflow itself. To limit biological variability, each method was applied to plasma derived from the same healthy donor. Proteomics analysis of the 24 resulting samples was conducted using an Astral mass spectrometer, with quality control HeLa samples (HeLa Pierce; ref 88329; Thermo Fisher Scientific) included prior to and after the batch to validate stable LC-MS/MS performances. When processing the raw data, the MBR was disabled to avoid artificial inflation of protein identifications across workflows. The comparative analysis assessed several performance criteria, including the identified protein landscape, dynamic range of identified proteins, repeatability, quantification bias, functional coverage, cost, and suitability for throughput applications.

**Figure 2:**
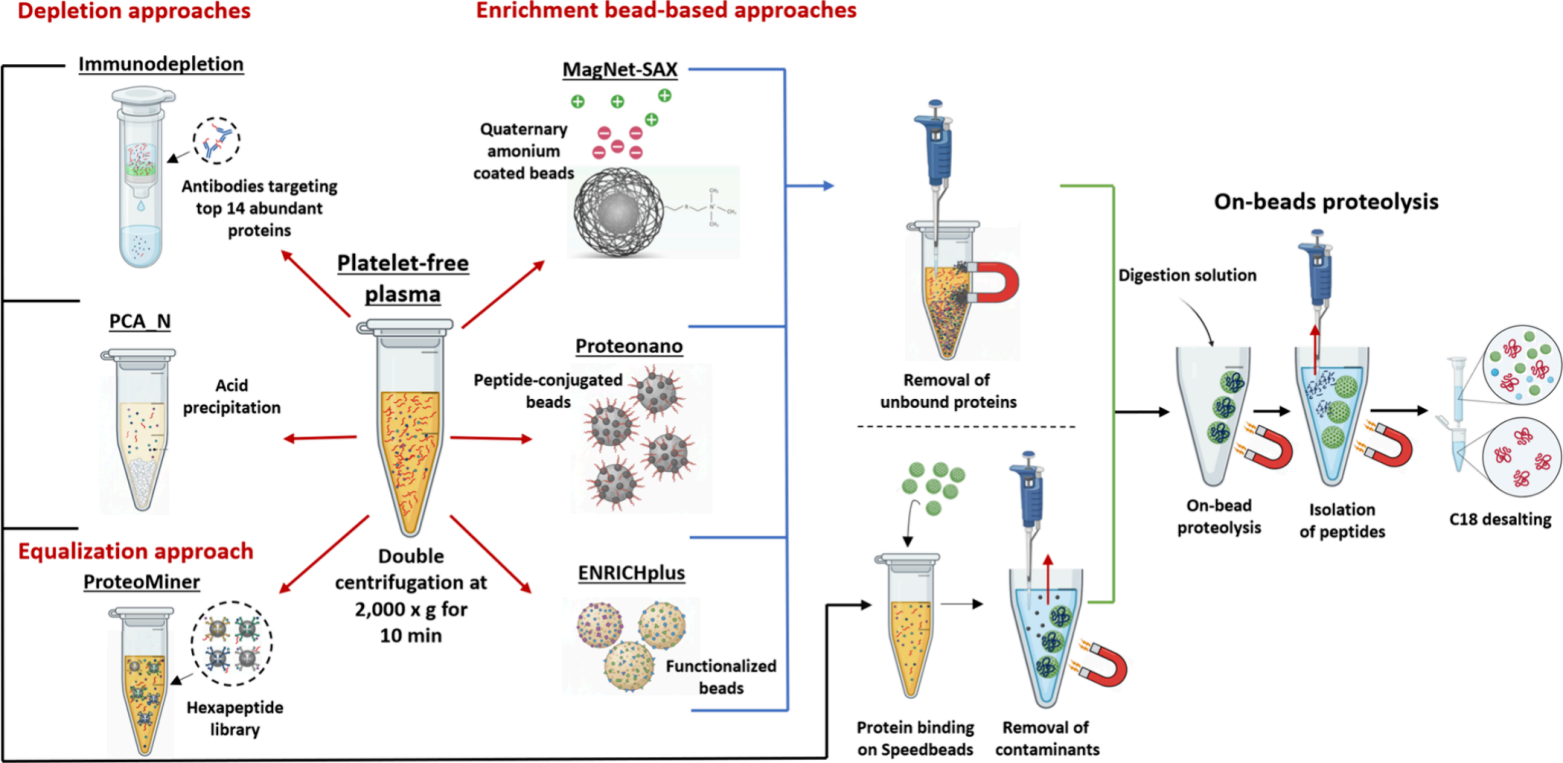
Overview of the experimental workflow for LC–MS/MS-based platelet-free plasma analysis.

### Identified Peptides and Protein Groups in Platelet-Free Plasma

Out of the seven methods, most are able to identify more peptides (Figure 3, panel A) and consequently more proteins (Figure 3, panel B) than the neat platelet-free plasma. However, two methods, namely PCA_N and ENRICHplus, do not allow to increase the peptide numbers while increasing the protein counts. Proteonano yielded the highest significant increase in proteome coverage (x2.1 fold) with 15,504 ± 531 peptides and 1955 ± 29 protein groups, relative to 8228 ± 157 peptides and 915 ± 12 protein groups identified in neat platelet-free plasma (*P* < 0.05). The mean peptide per protein ratio is 7.9 and 9.0, respectively. Immunodepletion ranked second for the number of protein groups identified, with a significant 1.9-fold increase, detecting 16,099 ± 34 peptides and 1747 ± 16 protein groups (*P* < 0.05). In this case, the mean peptide per protein ratio rises to 9.2. The remaining methods followed in descending order of significant enrichment of protein groups (*P* < 0.05): PM+ID, ProteoMiner, ENRICHplus and MagNet-SAX, which detected an average of 13,821 ± 599, 12,407 ± 688, 8134 ± 775, and 11,169 ± 262 peptides, corresponding to 1613 ± 54, 1420 ± 40, 1385 ± 144, and 1257 ± 26 protein groups, respectively. However, PCA-N showed an insignificant increase in the number of identified protein groups of 1094 ± 19 compared to 915 ± 12 in platelet-free neat plasma (*P* > 0.05). Most of these workflows showed very good consistency in the number of identified peptides and protein groups across replicates, except ENRICHplus, which displayed a higher standard variation of 144 protein groups and 775 peptides and had the lowest average peptide per protein ratio of 5.8.

**Figure 3:**
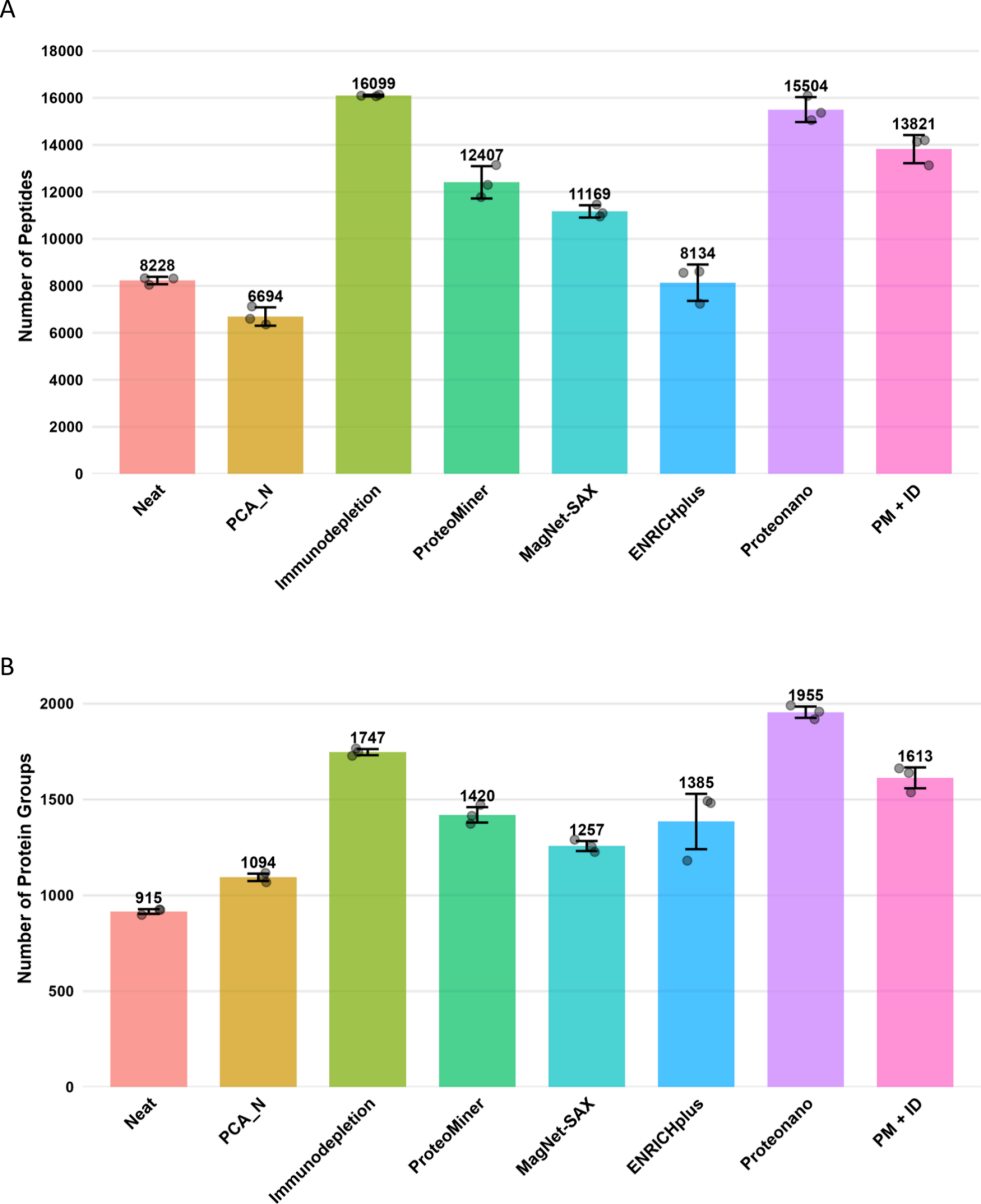

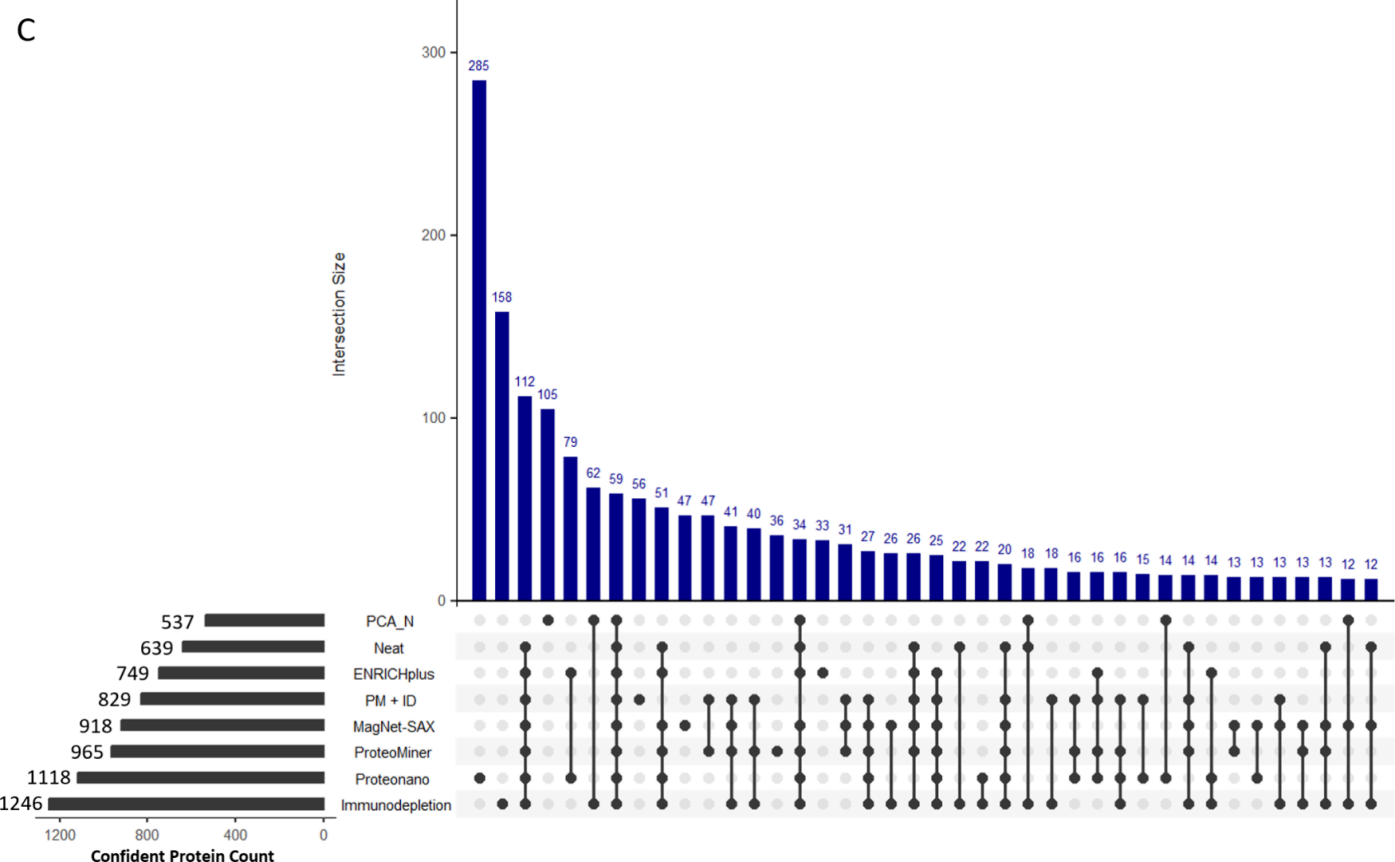
Average number of identified peptides (A) and protein groups (B) across three technical replicates. Bars represent the mean, and error bars indicate the standard deviation. Individual replicate values are shown as points. Upset plot displaying the 40 highest intersections of protein groups (C) for proteotypic proteins present in all technical replicates of each workflow with a %CV below 30%.

The percentage of protein groups identified with 1, 2, 3–5, 5–10, and more than 10 peptides for each workflow is shown in Figure S2, panel A. These percentages were calculated based on the peptides identified within each individual sample rather than globally across all samples as reported by the direct DIA-NN output. This approach ensures clarity about protein groups that may be supported by a different number of peptides across samples. Using this per-sample calculation, 76% of the protein groups identified with ProteoMiner were supported by at least two peptides. Lower proportions were observed for PCA_N (74%), MagNet-SAX (74%), Immunodepletion (72%), Proteonano (71%), Neat (69%), PM+ID (69%), and ENRICHplus (67%), respectively. In addition, 79 nonproteotypic protein groups were identified among the 3,220 protein groups in this study. The percentage of nonproteotypic protein groups for each workflow, in ascending order: PCA_N (1.9%), Immunodepletion (2.3%), ENRICHplus (2.4%), Proteonano (2.5%), MagNet-SAX (2.5%), Neat (2.5%), PM+ID (2.5%), and ProteoMiner (2.8%) (Figure S2, panel B).

The coefficient of variation (CV) across replicates was calculated, and the proteotypic protein groups identified in all the replicates of the workflow, with a %CV below 30% were denoted as “confident proteins.” Figure 3, panel C, shows the overlaps between methods in terms of coverage of these confident proteins. Immunodepletion, Proteonano, ProteoMiner, MagNet-SAX, PM+ID and ENRICHplus resulted in 1246, 1118, 965, 918, 829, and 749 confident proteins compared to 639 in the Neat, respectively. Conversely, PCA_N identified 537 confident proteins; 19% lower than the neat. Proteonano, Immunodepletion and PCA_N uniquely identified 285, 158, and 105 confident proteins that were not detected in the remaining workflows. For better clarity of the overlap between specific workflows, Venn diagrams are presented in Figure S3. Among the confident protein groups identified across the Neat, Immunodepletion, and Proteonano workflows, Immunodepletion uniquely identified 447 confident protein groups, compared with 515 confident protein groups uniquely identified by Proteonano. In addition, Immunodepletion demonstrated greater coverage of the neat proteome, sharing 590 confident protein groups with the neat sample, whereas Proteonano shared only 394 confident protein groups (Figure S3, panel A). With respect to the bead-based protein enrichment workflows, 365 confident protein groups were shared among ENRICHplus, MagNet-SAX, and Proteonano (Figure S3, panel B). Proteonano uniquely identified 423 confident protein groups, compared with 303 and 78 protein groups uniquely identified by MagNet-SAX and ENRICHplus, respectively. The combined ProteoMiner-Immunodepletion workflow confidently identified 105 protein groups that were not detected by either ProteoMiner or Immunodepletion alone. However, this gain came at the cost of 446 and 94 protein groups that were confidently identified when using Immunodepletion or ProteoMiner individually (Figure S3, panel C).

### Functional Analysis of Identified Proteins

According to the GO molecular function enrichment analysis of proteins in Figure 3, panel C, the confident proteins uniquely identified by immunodepletion are mainly associated with catalytic activities (Table S2). In contrast, the unique confident proteins identified by PCA_N exhibit protein binding and signaling functions. Additionally, the proteins uniquely identified by Proteonano are primarily involved in protein binding, along with catalytic activity and signaling functions.

GO functions assigned to the proteins that were consistently identified in all of the replicates revealed distinct enrichments. Figure 4, panel A, shows the four main represented functions: immunoglobulin complex, immune response, extracellular vesicle (EV) and blood coagulation, while results for the low-abundant proteins belonging to chemokines, cytokines, growth factors, and hormones are indicated in Figure 4, panel B. ProteoMiner demonstrated stable preservation of immunoglobulin complexes of 93 proteins, matching the number identified in the neat plasma. In contrast, fewer immunoglobulin complexes were detected in MagNet-SAX, PM+ID, immunodepletion, ENRICHplus, and PCA_N, yielding 84, 82, 77, 73, and 64 proteins, respectively. Interestingly, although Proteonano showed no significant immunoglobulin complex function, it facilitated the second-highest annotation of immune response function with 376 proteins after immunodepletion (384 proteins) compared to 263 proteins annotated in the neat. Also, PM+ID (314 proteins), ProteoMiner (292 proteins), PCA_N (284 proteins), ENRICHplus (278 proteins), and MagNet-SAX (276 proteins) showed an increase in the number of immune response proteins compared to the neat.

**Figure 4:**
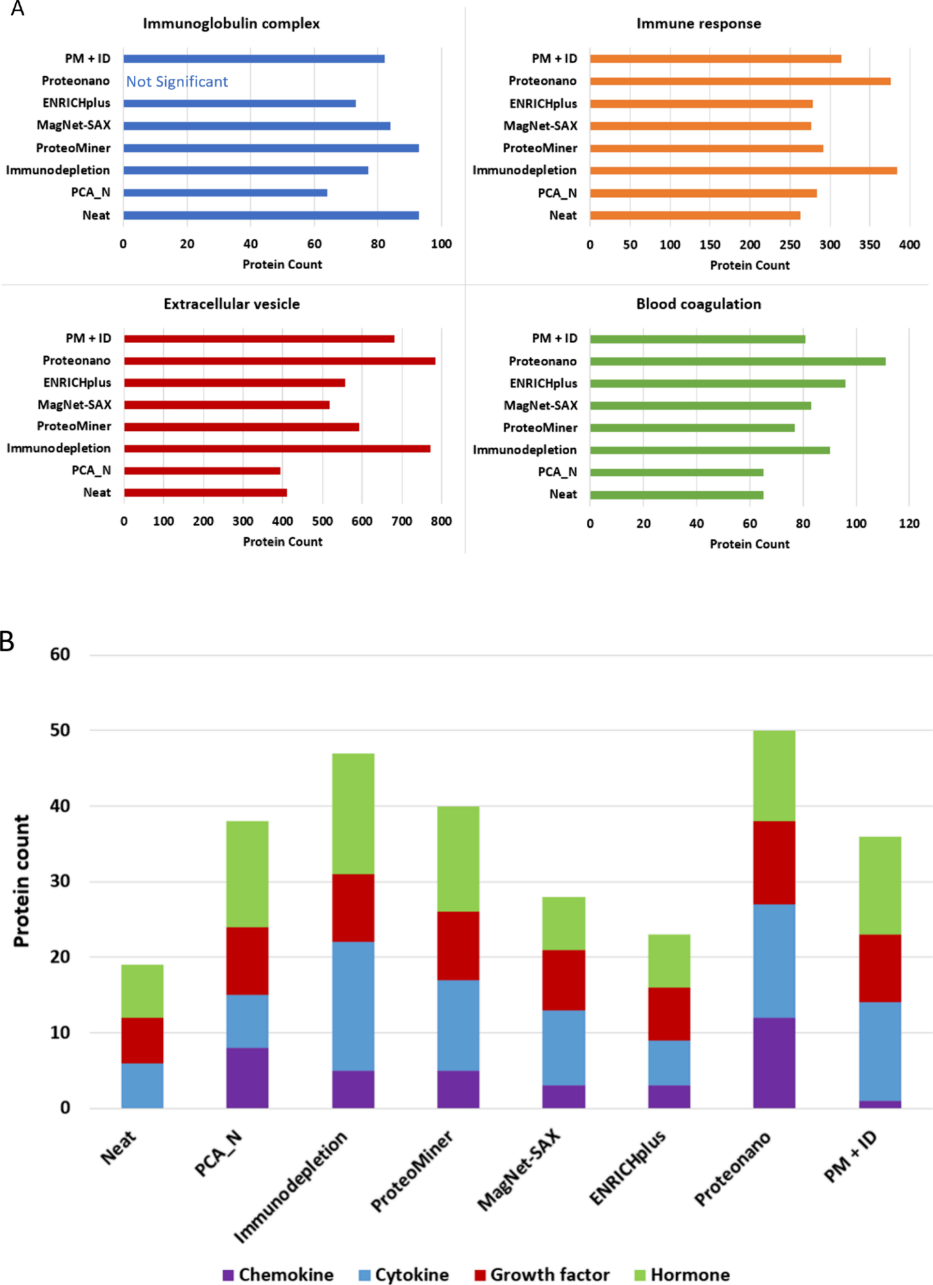
Functional analysis of identified proteins based on: the GO term enrichment using g:Profiler (A) and Human protein atlas (B).

Proteonano and immunodepletion enriched the highest number of proteins associated with EVs, identifying 784 and 772 proteins, respectively. In decreasing order, we found 681, 592, 558, and 519 consistently identified proteins by PM+ID, ProteoMiner, ENRICHplus, and MagNet-SAX, respectively, compared to 410 in the neat plasma, corresponding to a 2-fold increase in the enriched EVs between Proteonano and neat plasma. To further validate the presence of proteins possibly derived from EVs, we assessed the quantification of established EV reference markers, including CD9, TSG101, FLOT1, ITGAX, EZR, PDCD6IP, ITGAM, and SDCBP (Table S3). In regard to the quantification of EV markers identified, the workflows ranked in descending order as follows: Proteonano, ENRICHplus, ProteoMiner, PM+ID, and Immunodepletion, all of which displayed EV enrichment. In contrast, MagNet-SAX showed poor EV enrichment, and PCA_N showed no evidence of EV marker enrichment.

Blood coagulation proteins identified with every workflow was preserved at 65 proteins between PCA_N and neat, showing no enrichment of coagulation proteins. However, in descending order of enrichment, Proteonano (111 proteins), ENRICHplus (96 proteins), Immunodepletion (90 proteins), MagNet-SAX (83 proteins), PM+ID (81 proteins), and ProteoMiner (77 proteins) showed a considerable increase in the number of blood coagulation proteins. These results correlate with the sum of the blood coagulation proteins’ abundances in each workflow (Figure S4). In addition, GO analysis significantly annotated 65 and 53 platelet activation-related proteins in the Proteonano and ENRICHplus workflows, respectively, whereas no significant functional enrichment of platelet activation proteins was observed for the remaining workflows. Uniquely, Proteonano significantly annotated 27 proteins to have a role in G protein function, while Immunodepletion and PCA_N annotated 61 and 47 cell adhesion proteins, respectively (Table S4). The complete list of uniquely annotated clusters across workflows is presented in Figure S5. Interestingly, Proteonano resulted in the enrichment of a larger number of GO terms compared to the other workflows. These enriched terms covered diverse functional categories, highlighting broader functional coverage rather than enrichment confined to a specific pathway or process.

The ability of the workflows to identify very low-abundant proteins was assessed by matching the identified proteins with cytokines, chemokines, growth factors, and hormones reported in the blood secretome cluster of the Human Protein Atlas (Figure 4, panel B). Overall, Proteonano enriched the highest number of proteins from these clusters followed by immunodepletion, identifying 50 and 47 proteins, respectively. ProteoMiner, PCA_N, PM+ID, MagNet-SAX and ENRICHplus enriched these clusters in a lower ratio, identifying 40, 38, 36, 28, and 23 proteins, compared to 19 in neat plasma. Remarkably, all the workflows yielded the identification of chemokines that were not identified by the analysis of the neat plasma. Interestingly, the order of the workflows changes when considering only the confident proteins: Immunodepletion (40 proteins), Proteonano (32 proteins), ProteoMiner (31 proteins), PCA_N (29 proteins), MagNet-SAX (25 proteins), PM+ID (24 proteins), ENRICHplus (17 proteins), and Neat (12 proteins) (Figure S6).

### Dynamic Range of Plasma Proteome Analysis

The possible dynamic range of plasma proteome analysis achieved by each workflow was assessed by mapping the identified proteins to their corresponding known blood concentrations (pg/L), as reported in the Human Protein Atlas. Unfortunately, not all identified proteins could be taken into consideration as 15,868 proteins lacked mass spectrometry-based annotation of blood concentration in the Atlas. For example, the numbers of proteins without blood concentration annotation were 39, 64, 56, 61, 44, 57, 174, and 84 in Neat, PCA_N, Immunodepletion, ProteoMiner, MagNet-SAX, ENRICHplus, Proteonano, and PM+ID, respectively. Protein concentrations were classified into seven ranges, from the lowest (<10^5^ pg/L) to the highest (>10^10^ pg/L) range. The dynamic range analysis was conducted on proteins identified across all replicates (Figure 5, panel A) and confident proteins (CV < 30%) identified across all replicates (Figure 5, panel B).

**Figure 5:**
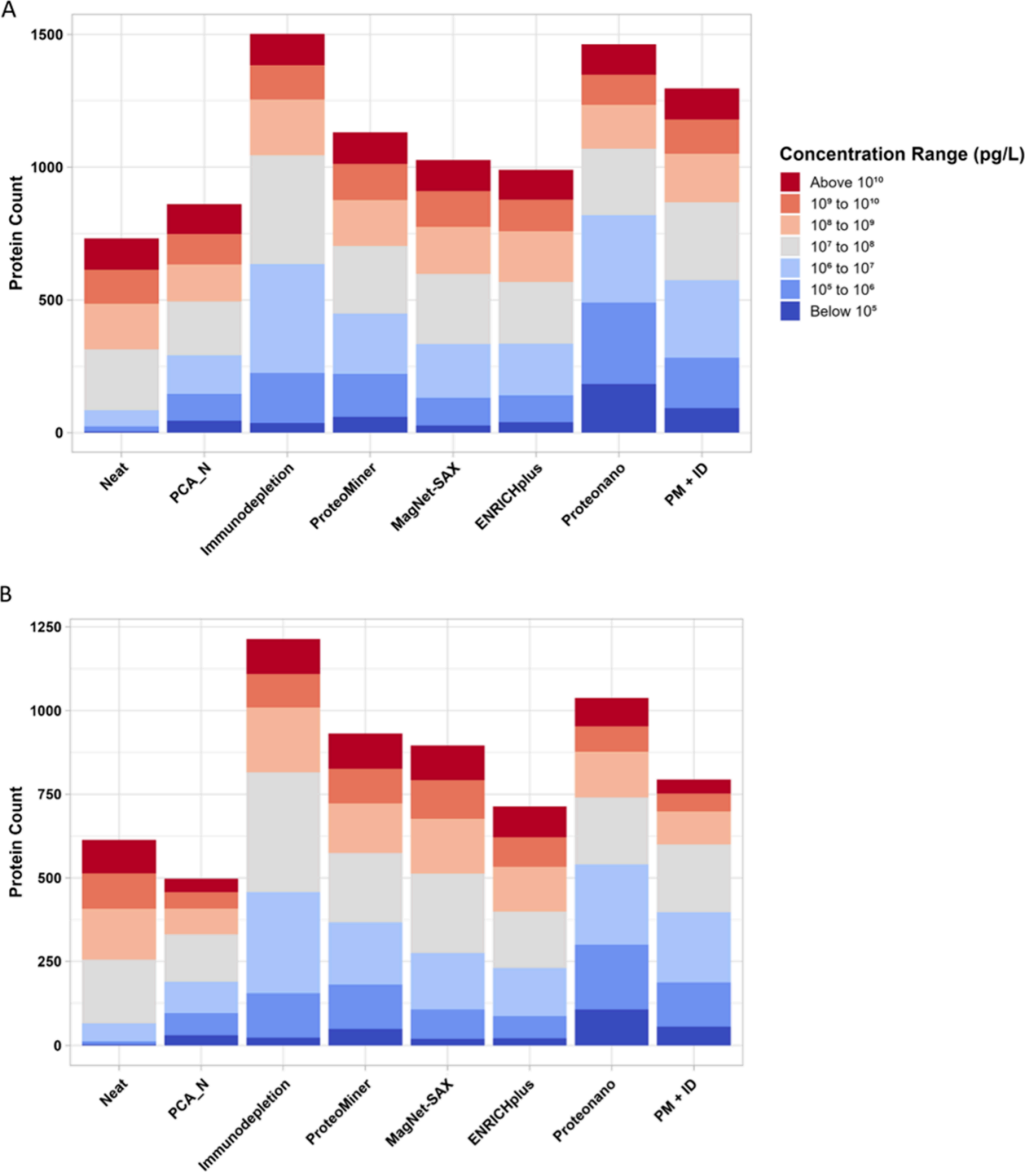
Depth of proteome analysis presented as (A) concentration of proteins identified in all replicates of each workflow. (B) Confident proteotypic proteins identified in all replicates with a %CV below 30%. Protein concentrations are based on the Human Protein Atlas mass spectrometry data and classified into seven concentration ranges: below 10^5^, 10^5^ to 10^6^, 10^6^ to 10^7^, 10^7^ to 10^8^, 10^8^ to 10^9^, 10^9^ to 10^10^, and above 10^10^ pg/L.

Considering the proteins consistently identified in all replicates of every workflow, high abundance proteins (>10^8^ pg/L) were enriched up to 457, 430, 429, 428, and 423 proteins using immunodepletion, PM+ID, MagNet-SAX, ProteoMiner, and ENRICHplus, respectively, compared to 418 proteins detected unde the neat condition. In contrast, both Proteonano and PCA_N demonstrated slightly lower detection of proteins known as high abundance, identifying 393 and 367 proteins, respectively.

For moderate abundance proteins (10^6^–10^8^ pg/L), immunodepletion yielded the highest coverage (820 proteins), followed by PM+ID (585 proteins) and Proteonano (580 proteins). ProteoMiner (481 proteins), MagNet-SAX (466 proteins), and ENRICHplus (426 proteins) identified similar numbers of proteins, whereas PCA_N (348 proteins) showed a marked decrease compared to neat plasma (290 proteins).

Concerning the low-abundant proteins (<10^6^ pg/L), the workflows exhibited varying capacities of detection. The numbers of proteins identified are ranked in descending order as follows: Proteonano (490 proteins), PM+ID (282 proteins), Immunodepletion (225 proteins), ProteoMiner (222 proteins), PCA_N (146 proteins), ENRICHplus (141 proteins), and MagNet-SAX (132 proteins), all showing an improved depth of proteome coverage relative to neat plasma (24 proteins).

The analysis of the confident proteotypic proteins (%CV < 30%) at the high-abundant protein’s concentration ranges (>10^8^ pg/L) revealed similar values for immunodepletion (399 proteins), MagNet-SAX (382 proteins), Neat (359 proteins), and ProteoMiner (358 proteins) (Figure 5, panel B). While ENRICHplus (313 proteins) and Proteonano (297 proteins) displayed slightly lower identifications, PM+ID (194 proteins) and PCA_N (166 proteins) showed a drastically lower number of confident high-abundant proteins.

For moderate abundance proteins (10^6^–10^8^ pg/L), immunodepletion (660 proteins) demonstrated the highest coverage of confident proteins, followed by Proteonano (442 proteins), PM+ID (413 proteins), MagNet-SAX (406 proteins), and ProteoMiner (393 proteins), whereas ENRICHplus (313 proteins), Neat (243 proteins), and PCA_N (236 proteins) covered a narrower range of confident moderate abundance proteins.

At low abundance protein ranges (<10^6^ pg/L), the descending order of confident protein identifications was: Proteonano (299 proteins), PM+ID (187 proteins), ProteoMiner (181 proteins), Immunodepletion (155 proteins), MagNet-SAX (107 proteins), PCA_N (96 proteins), ENRICHplus (87 proteins), and Neat (12 proteins).

### Protein Enrichment and Precision Assessment

The precision of the measurements was assessed through the %CV distribution and the median CV across the proteins that are consistently identified in all of the replicates of each workflow (Figure 6, panel A). The ranking in ascending order of median CV is as follows MagNet-SAX (13%), ProteoMiner (15%), Immunodepletion (16%), ENRICHplus (21%), Proteonano (21%), PCA_N (24%), and PM+ID (24%). Notably, a higher median CV value does not explicitly correspond to lower number of confident proteins. As highlighted in Figure 3, panel C, immunodepletion identified 1246 proteotypic proteins with low CV (<30%), compared to 918 proteins identified using MagNet-SAX.

**Figure 6:**
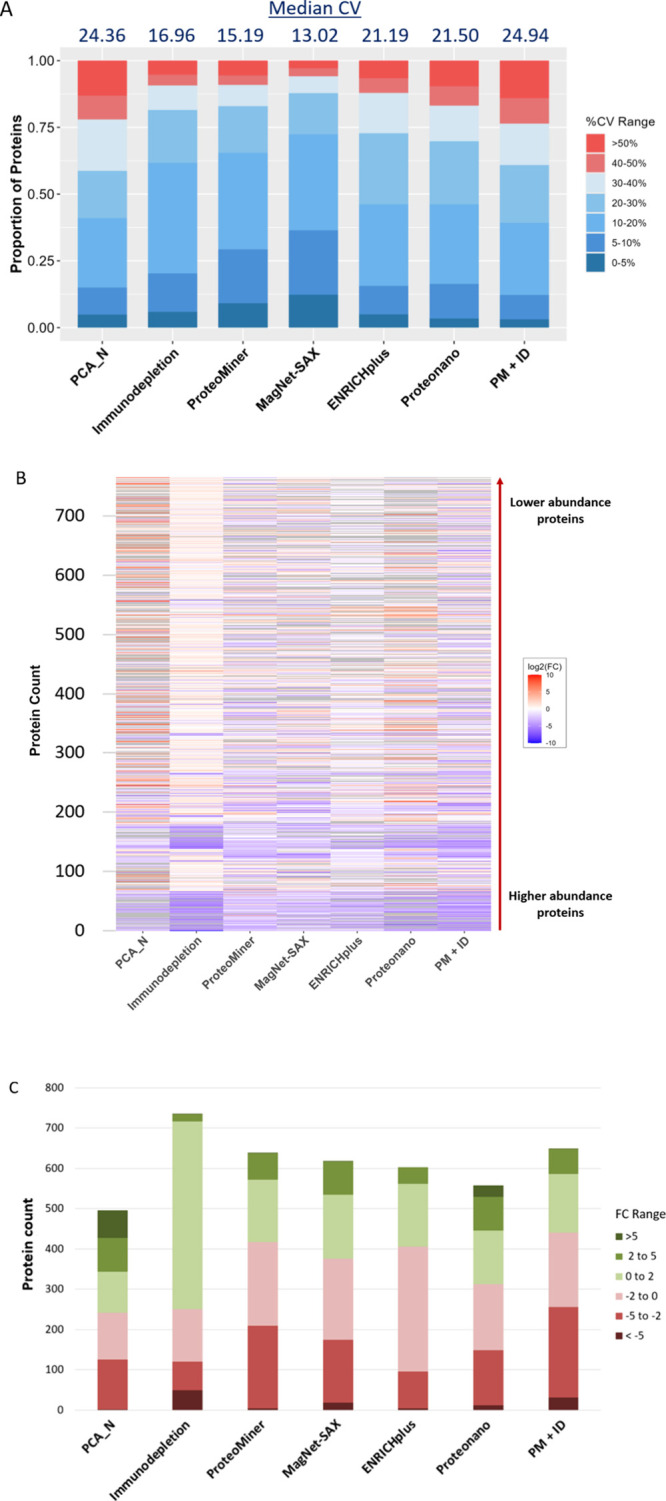
(A) Distribution of %CV for proteins consistently identified in the three replicates of each workflow, classified into seven %CV ranges: 0–5%, 5–10%, 10–20%, 20–30%, 30–40%, 40–50%, and >50%. (B) Average fold change in protein quantification (relative to the corresponding measurement in the neat plasma replicates), calculated across the three replicates, for enriched (red), depleted (blue), and missing (gray) proteins in each workflow. (C) Grouping of the proteins fold change values from (B) into five ranges: < −5, −5 to −2, −2 to 2, 2 to 5, and >5. Panels B and C focus on proteins common to the neat plasma condition (771 proteins). %CV values are based on label-free quantification (LFQ), while fold change analyses are performed on log-transformed protein quantification data.

The protein enrichment factor was evaluated for each method by calculating the fold change of each protein’s abundance relative to its quantification under the neat condition, as detailed in the Data Interpretation and Statistical Analysis section. This analysis included only proteins consistently identified in the neat samples. Figure 6, panel B, shows these fold changes for proteins ranked from the most to the least abundant in the neat plasma from bottom to top. As expected, immunodepletion achieved a notable depletion of high abundance proteins, reaching up to a 10-fold reduction on a logarithmic scale (log2), yet showed only minimal enrichment of moderate abundance proteins when compared to the neat. In comparison, ProteoMiner, ENRICHplus, and MagNet-SAX also depleted high abundance proteins (2-fold), but these reductions were less pronounced than those observed with immunodepletion, accompanied by inconsistent patterns of enrichment and depletion among moderate abundance proteins. Similarly, PCA_N depleted high abundance proteins (2-fold) to the same extent but exhibited a higher degree of inconsistent enrichment of moderate abundance proteins, reaching up to 5-fold compared to the Neat condition. PM+ID and Proteonano depleted high abundance proteins to a higher extent (4-fold). Proteonano exhibited inconsistent behavior with moderate abundance proteins, showing enrichment up to 5-fold alongside pronounced depletion up to 5-fold. In contrast, PM+ID demonstrated a lower extent of enrichment for moderate abundance proteins (around 2-fold) but a stronger depletion effect, reaching up to 5-fold.

For better assessment of the number of proteins depleted and enriched using the various technologies, the FC values of the proteins from Figure 6, panel B, are grouped into FC ranges: drastic depletion (FC ≤ −5), moderate depletion (−5 < FC ≤ −2), minimal deviation (−2 < FC ≤ 2), moderate enrichment (2 < FC ≤ 5), and drastic enrichment (FC > 5), presented in Figure 6, panel C.

Apart from the protein abundance, the highest number of highly depleted proteins (FC ≤ −5), was demonstrated by Immunodepletion and PM+ID, drastically depleting 49 and 31 proteins, respectively. Followed by MagNet-SAX and Proteonano depleting, 18 and 12 proteins, respectively, at more than 5-fold reduction. However, ProteoMiner, ENRICHplus and PCA_N only 4, 4, and 1 protein, respectively, at this severe depletion. At a lower proximity of depletion (−5 < FC ≤ −2), PM+ID, ProteoMiner, MagNet-SAX, Proteonano, PCA_N, ENRICHplus, and Immunodepletion depleted 225, 205, 157, 137, 124, 92, and 71 proteins, respectively.

For proteins minimally deviating from Neat plasma (−2 < FC ≤ 2), Immunodepletion conserved the quantification of 596 proteins followed by ENRICHplus, ProteoMiner, MagNet-SAX, PM+ID, Proteonano and PCA_N with 466, 363, 359, 330, and 218 proteins, respectively.

Concerning moderately enriched proteins (2 < FC ≤ 5), PCA_N, Proteonano, MagNet-SAX, ProteoMiner, PM+ID, and ENRICHplus enriched for 85, 84, 83, 66, 62, and 41 proteins, respectively. By contrast, immunodepletion enriched for 18 proteins in this protein enrichment range. At higher protein enrichment levels (FC > 5), PCA_N and Proteonano drastically enriched the quantification of 68 and 28 proteins, respectively. Severe enrichment was less frequent with immunodepletion (2 proteins), ProteoMiner (1 protein), MagNet-SAX (1 protein), PM+ID (1 protein), and not at all for ENRICHplus.

The standard deviation of the fold change (FC SD) between replicates, corresponding to the standard deviation of log2FC values obtained from replicates of each workflow, is reported in Figure S7, panel A, once again for proteins ranked from the most abundant to the least abundant in the neat plasma. These values of variability in quantification values were grouped into defined ranges, and the corresponding median FC SD for each workflow was calculated (Figure S7, panel B). The ranking in ascending order of the precision of measurements across replicates is as follows: MagNet-SAX (16%), ProteoMiner (20%), Immunodepletion (21%), Proteonano (26%), ENRICHplus (29%), PM+ID (37%), and PCA_N (38%). However, not all proteins identified under the neat condition were detected across the various workflows, as reflected by differences in the numbers of proteins to be considered, indicating variable proteome coverage relative to Neat. Considering only proteins consistently identified in all replicates, the proteome coverage relative to the neat condition was ranked as follows: Immunodepletion demonstrated the highest coverage at 95%, followed by PM+ID at 84%, ProteoMiner at 82%, MagNet-SAX at 80%, ENRICHplus at 78%, Proteonano at 72%, and PCA_N showing the lowest coverage at 64%.

### Pairwise Correlation and Distribution of Protein Quantifications across the Different Methods

Pairwise correlation analysis of confident protein quantification was performed, considering only proteotypic proteins shared across specified workflows with a %CV below 30%. Thus, the pairwise correlations include a distinct number of proteins, depending on the confident protein overlap between workflows. Compared to the neat plasma, the variation in quantification between workflows (Figure 7) was consistent with the fold change analysis (Figure 6, panel B). Among the tested workflows, ENRICHplus demonstrated the highest correlation with the neat (93%), followed by PM+ID (90%), Immunodepletion (89%), ProteoMiner (88%), MagNet-SAX (86%), and Proteonano (78%). The lowest correlation with neatness was observed with PCA_N (72%). Notably, PCA_N also showed the weakest correlations with the other workflows: Immunodepletion (75%), ENRICHplus (71%), Proteonano (67%), MagNet-SAX (66%), PM+ID (64%), and ProteoMiner (63%). In contrast, the strongest association between protein depletion and enrichment workflows was found between PM+ID and ProteoMiner, which showed nearly complete correlation (99%) followed by Proteonano and ENRICHplus (92%).

**Figure 7:**
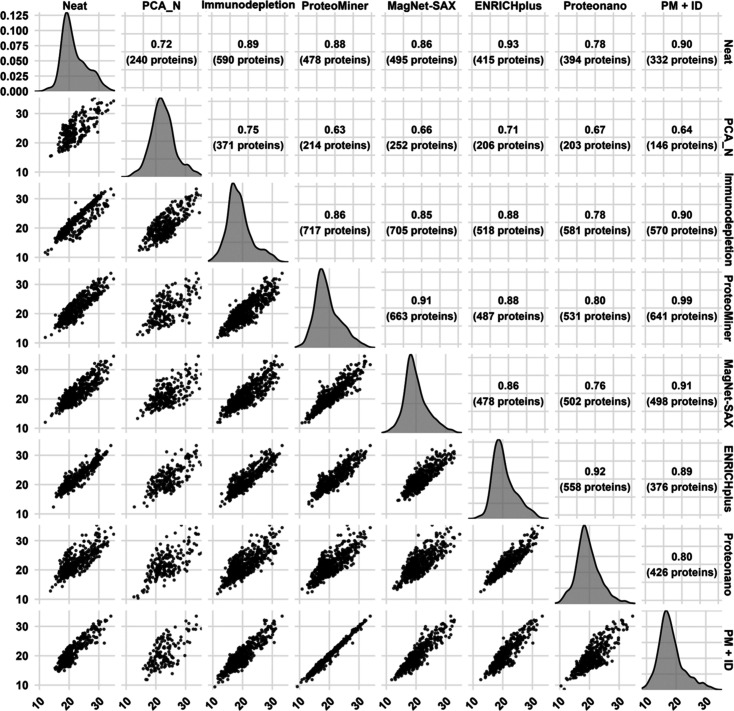
Pairwise Pearson correlation and distribution of protein quantifications across plasma proteome workflows. The correlation plot focuses on confident proteins common to the specified workflows; specified between parentheses.

### Stability of Proteonano-Conjugated Peptides during Tryptic Digestion

Proteonano beads are conjugated with three peptides: HKAATKIQASFRGHITRKKLC, DIEEVEVRSKYFKKNERTVEC, and QETLKDTRSKFFNKPSMTVVC. These peptides contain lysine and arginine residues and could be susceptible to tryptic digestion. To verify that these conjugated peptides were not released or cleaved during proteolysis, we examined all theoretical tryptic peptides derived from these sequences and assessed their taxonomical origins using BLASTp. Only one subsequence (IQASFRGHITRK) showed a potential match to a human protein (neuromodulin, GAP43; sp|P17677|NEUM_HUMAN). However, neither this peptide nor its parent sequence was identified in the proteome data set of Proteonano or any other evaluated workflow. Furthermore, a dedicated DIA-NN search was performed using the *Homo sapiens* database supplemented with these three Proteonano-conjugated peptide sequences, and no precursor identifications originating from these peptides were detected.

## Discussion

Plasma proteomics has been deployed for the identification of biomarkers of diseases,^39–41^ relying on the ease of sample handling, low invasiveness, and the diversity of proteins originating from several organs. Although plasma is rich in more than 20,000 proteins, only 22 proteins represent 99% of the plasma content.^2^ This wide dynamic range of plasma proteins limits the detection of low abundance proteins, which requires preanalytical processing of plasma to tackle this dynamic range. Another challenge is the ex vivo activation of platelets, which causes an inflation of the number of proteins identified.^13^ To limit the effect of platelets on the results of the present study, we restricted the analysis to platelet-free plasma prepared by double centrifugation at 2,000*g* for 10 min.^21^ We systematically benchmarked several protein enrichment or depletion workflows, including PCA_N, MagNet-SAX, ENRICHplus, Immunodepletion, Proteonano, ProteoMiner, and the consequent combination of ProteoMiner followed by Immunodepletion. To ensure accurate comparability and repeatability if a single workflow is adopted, all analyses excluded MBR without the addition of missing values.

Our results demonstrated the strength of Proteonano and Immunodepletion which doubled the number of identified protein groups compared to the neat platelet-free plasma. This enrichment persisted at the level of confident proteins that are consistently identified in all replicates with a low CV (<30%), as shown in Figure 3, panel C. PM+ID, ProteoMiner, ENRICHplus, and MagNet-SAX yielded relatively lower enrichment of proteins (1.3–1.7 folds); however, PCA-N showed no significant enrichment. While direct comparisons with previous studies are limited by differences in plasma preparation and LC–MS/MS parameters, the number of proteins identified with PCA_N (∼1000 proteins) aligns with a previously published study that used centrifugation at 3000 × g for 7 min.^13^ In contrast, applying a different centrifugation protocol (2000 × g for 20 min) resulted in a higher number of proteins detected with PCA_N (∼1300 proteins) compared to neat plasma (∼630 proteins) analyzed.^8^ Although that work employed a shorter LC–MS/MS gradient (11.5 min) than in the present study, the increased protein yield can be related primarily to the plasma centrifugation procedure and the platelet content inflating the number of proteins identified.^13^

Interestingly, Immunodepletion showed the highest overlap (95%) of proteins consistently identified in the platelet-free neat plasma, followed by PM+ID (84%), ProteoMiner (83%), MagNet-SAX (80%), ENRICHplus (78%), Proteonano (72%) and PCA_N (64%), which further supports the extension of the proteome identified in the neat plasma. Overall, these results are consistent with previous studies reporting the ability of these workflows to enrich plasma proteins. However, distinct enrichment performance has been reported by previous studies utilizing different plasma preparation protocols and thus different platelet contents. For instance, ENRICHplus identified around 2100 proteins^23^ when plasma was prepared by single centrifugation at 3000*g* for 5 min, and 2900 proteins^27^ when prepared by double centrifugation at 1500*g* for 10 min, suggesting that plasma preparation workflows have a considerable impact on the observed proteomes. As reported previously, parameters such as the type of anticoagulant used during plasma preparation, centrifugation speed, and rounds highly influence the platelet composition of the plasma samples, thus impacting the proteomics workflow.^13^ Additionally, the different analytical workflows also contribute to the interstudy variability.

Distinct profiles of protein functional enrichment were highlighted by various workflows. Proteonano and Immunodepletion yielded a 2-fold enrichment of EVs compared to the neat plasma. ProteoMiner completely preserved the identification of immunoglobulin complex proteins, which were depleted up to 1.4-fold in PCA_N. In Proteonano, although the proteins were detected, the corresponding GO enrichment did not reach significance. In contrast, Immunodepletion and Proteonano showed a 1.4-fold increase in proteins associated with the immune response compared with neat plasma. ENRICHplus and MagNet-SAX yielded an intermediate enrichment of EVs, immunoglobulin complex, and immune response proteins. In addition, proteonano- and Immunodepletion enriched the identification and quantification of blood coagulation proteins. These proteins require careful monitoring in plasma proteomics, as their presence may reflect either their biological significance in clinical studies or effects of sample handling.^42^ Notably, both workflows captured this protein class. In addition, Proteonano and ENRICHplus captured platelet activation–related proteins, even in platelet-free plasma, where only minimal traces of activated platelets are expected to be present.

At the level of cytokines, chemokines, growth factors, and hormones, Proteonano showed optimal enrichment of 50 proteins consistently identified in the replicates, followed by Immunodepletion enriching 48 proteins compared to 18 proteins in the platelet-free neat plasma. However, when considering confident protein groups, only 30 proteins were consistently identified for Proteonano, indicating slightly lower repeatability compared to 40 confident proteins for Immunodepletion. The ability of Proteonano to enrich low-abundant proteins (<10^6^ pg/L) was further demonstrated by consistently identifying 490 proteins, of which 299 proteins had a low CV (<30%) compared to 12 proteins in the neat plasma. Immunodepletion substantially enriched for moderate abundance proteins, further extending coverage beyond neat plasma. Notably, the higher number of enriched GO terms observed with Proteonano is not solely attributable to the total number of identified proteins but rather to differences in proteome composition. Although immunodepletion yielded a comparable number of proteins, Proteonano enabled the detection of a greater proportion of low abundance proteins. These proteins are often involved in regulatory, signaling, and compartment-specific biological processes, and their recovery increases the diversity of functional annotations captured in enrichment analyses. Interestingly, the uniquely identified proteins by Proteonano were primarily associated with signaling, binding, and G protein-coupled activity, whereas those unique to Immunodepletion are mainly involved in catalytic activities. PCA_N, despite its limited enrichment effect, produced a distinct proteome profile from the neat platelet-free plasma, enriching for cell adhesion proteins and capturing 146 low abundance proteins mainly annotated to binding and signaling functions (Tables S2 and S4, Figures 3(panel C) and 5). Our results differ from previous studies reporting the enrichment of EVs using MagNet-SAX^10^ as no considerable identification of EV reference markers was established here (Table S3)^43^; however, this divergence is supported by a recent study,^27^ showing the importance of multiple interlaboratory studies for plasma proteomics robust comparisons.^44^

Protein quantification enrichment factor and precision also varied across the approaches. ENRICHplus achieved a minimal deviation from the neat. Immunodepletion effectively depleted high-abundant proteins (∼10-fold) while maintaining relatively stable quantification of the remaining proteome. PM+ID, ProteoMiner, ENRICHplus, and MagNet-SAX showed more variable deviation, while Proteonano and PCA_N induced larger deviations in protein quantification. Consistent with these results, correlation analyses confirmed that ENRICHplus resembles neat platelet-free plasma (92% correlation). Assessment of quantification precision across replicates revealed that MagNet-SAX was most robust based on the percentage of identified proteome, followed by ProteoMiner, Immunodepletion, Proteonano, and ENRICHplus, whereas PM+ID and PCA_N showed lower precision (Figure S7, panel B). However, considering the number of confidently identified proteins with a low CV (<30%), Immunodepletion and Proteonano excelled, followed by ProteoMiner, MagNet-SAX, PM+ID, ENRICHplu,s and PCA_N (Figure 3, panel C). These results correlate with the previously reported results concerning the lower precision of PCA_N and ENRICHplus compared to the other workflows.^27,23^

The PCA_N and MagNet-SAX methods are the cheapest at approximately 2 and 10 euros per sample, respectively. Moderate-cost workflows such as ProteoMiner, Immunodepletion, and Proteonano were approximately 50 euros per sample. Moreover, the combined PM+ID strategy and the ENRICHplus kit were more expensive, at 100 and 150 euros per sample, respectively. Notably, we could not include in the benchmark the Proteograph methodology because of its cost per sample and the need of a specific automate. Apart from ProteoMiner, the evaluated technologies have potential for automation, which enhances their suitability for throughput workflows and improved repeatability. However, all evaluated methods were manually performed in our experimental setup.

In conclusion, all of the evaluated technologies effectively reduce the dynamic range of the plasma proteome, enabling deeper protein analysis. In this study, the best results were achieved using Proteonano and Immunodepletion, targeting low and moderately abundant proteins, respectively, while MagNet-SAX demonstrated the highest precision. The sequential combination of ProteoMiner and immunodepletion led to deeper analysis but at the expense of throughput and repeatability. Our results further indicate that the choice workflow can be tailored to the specific functional set of targeted proteins. Considering the limited agreement across studies on the performance of these workflows, mainly due to differences in plasma preparation protocols, preliminary testing is recommended when they are applied to plasma prepared using protocols that have not been previously evaluated. Future work should focus on standardizing plasma preparation protocols to enhance the repeatability and comparability of enrichment technologies.

### Limitations of the Study

In this study, we systematically evaluated and compared multiple protein depletion and enrichment workflows applied to platelet-free plasma, assessing their proteomic depth, protein enrichment, precision, and functional coverage. However, several experimental considerations should be acknowledged. All workflows were evaluated using a single plasma sample analyzed in technical replicates. Therefore, the assessment primarily reflects the technical performance of each workflow for a unique biological condition, but the results and ranking of methods may vary depending on the plasma sample. In addition, workflow-specific postenrichment processing strategies were applied. SP3 digestion was used for the Neat, PCA_N, Immunodepletion, ProteoMiner, and PM+ID workflows, whereas bead-based enrichment approaches (Proteonano, ENRICHplus, and MagNet-SAX) incorporated proteolysis directly on their corresponding enrichment beads. Although these differences may have influenced the results, the lack of a universally applicable postenrichment processing strategy across all workflows necessitated the use of workflow-optimized protocols. Notably, SP3 digestion most closely resembles bead-based proteolysis, as digestion also occurs directly on beads.^45^ Another critical consideration is that the same amount of SP3 beads was used to perform proteolysis for the Neat, PCA_N, Immunodepletion, ProteoMiner and PM + ID workflows. Because each workflow started with different plasma volumes and involved distinct processing strategies, the total protein output likely differed between the approaches. Consequently, the protein-to-bead ratio during SP3 digestion was not equivalent across workflows, which may have influenced the results.^45^ Additionally, trypsin was applied in the same amount across all workflows, irrespective of the total depleted or enriched protein content. While this standardized approach facilitated cross-workflow comparison, differences in protein load may have modestly influenced trypsin proteolysis efficiency and, consequently, the observed results.

This study additionally employed a prerelease version of the ENRICHplus kit, which may have undergone further optimization by the time of publication. Furthermore, plasma input volumes differed among the evaluated approaches, to comply with the manufacturer’s instructions. While the use of different plasma volumes could have influenced performance, comparable input volumes were applied for the bead-based workflows ENRICHplus (50 μL), MagNet-SAX (50 μL), and Proteonano (40 μL), thereby minimizing volume-related effects in their comparative analysis.

## Supplementary Material









## Data Availability

The mass spectrometry proteomics data have been deposited to the ProteomeXchange Consortium via the PRIDE partner repository with the data set identifier PXD069187.

## Notes

Figure S1: RBC and Coagulation contamination indices based on Baize software. Figure S2: Peptide-to-protein group and nonproteotypic protein groups percentages distribution across workflows. Figure S3: Overlap of confident protein groups identified across workflows. Figure S4: Summed abundances of coagulation proteins across workflows. Figure S5: GO term analysis for unique functions across workflows. Figure S6: Functional analysis of confident proteins based on the Human protein atlas. Figure S7: Fold change standard deviation of proteins across replicates of each workflow. Table S1: Platelet markers quantification in platelet-free plasma. Table S2: GO term analysis of the uniquely identified proteins across workflows. Table S3: Quantitative analysis of the EV markers across various approaches. Table S4: GO term analysis highlights of specific functions across workflows (PDF)
File 1—Comprehensive_Proteomics_Data (XLSX)
File 2—Tukey_Results_OWA_Figure_2 (XLSX)
File 3—Protein_Counts_by_Concentration (XLSX)
