# Supplementary material for: Systematic Evaluation of Depletion and Enrichment Technologies for Platelet-Free Plasma Proteomics
Source: J Proteome Res. 2026 Mar 17;25(6):2723–39. doi: 10.1021/acs.jproteome.5c01056 (PMC13248010; doi:10.1021/acs.jproteome.5c01056)
Supplement: Supplementary file 1 [file pr5c01056_si_001.pdf]

Systematic evaluation of depletion and enrichment technologies for platelet-free plasma proteomics

Salem Al Siblani[1, 2, 3], Jean Armengaud[1, 3], Clément Lozano[1, 3] *

[1] Institut Hospitalo-Universitaire Comprehensive SEPSIS Center, Paris-Saclay University, 91190 Saclay, France

[2] Département Médicaments et Technologies pour la Santé (DMTS), Université Paris-Saclay, CEA, INRAE, SPI, 30200 Bagnols-sur-Ceze,̀ France; Laboratoire Innovations Technologiques pour la Détection et le Diagnostic (Li2D), Université de Montpellier, F-30207 Bagnols-sur-Ceze,̀ France

[3] Université Paris-Saclay, CEA, INRAE, Département Médicaments et Technologies pour la Santé (DMTS), SPI, 30200 Bagnols-sur-Cèze, France

*Author to whom correspondence should be addressed: Clément Lozano, CEA-Marcoule, DRF/Joliot/DMTS/SPI/Li2D, "Innovative Technologies for Detection and Diagnosis" Laboratory, BP 17171, F-30200 Bagnols-sur-Cèze, France; clement.lozano@cea.fr

Keywords: Plasma proteomics, Platelet-free plasma, PCA-N, Immunodepletion, ProteoMiner, MagNet-SAX, ENRICHplus, Proteonano, ASTRAL, DIA.

Supporting Information

The following supporting information is available free of charge at the ACS website http://pubs.acs.org:

Figure S1 – RBC and Coagulation contamination indices based on Baize software

Figure S2 – Peptide-to-protein group and non-proteotypic protein groups percentages distribution across workflows

Figure S3 – Overlap of confident protein groups identified across workflows

Figure S4 – Summed abundances of coagulation proteins across workflows

Figure S5 – GO term analysis for unique functions across workflows

Figure S6 – Functional analysis of confident proteins based on the Human protein atlas

Figure S7 – Fold change standard deviation of proteins across replicates of each workflow

Table S1 – Platelet markers quantification in platelet-free plasma

Table S2 – GO term analysis of the uniquely identified proteins across workflows

Table S3. Quantitative analysis of the EV markers across various approaches

Table S4 – GO term analysis highlights of specific functions across workflows

File 1 – Comprehensive_Proteomics_Data.xlsx

File 2 – tukey_results_OWA_Figure_2.xlsx

File 3 – Protein_Counts_by_Concentration.xlsx

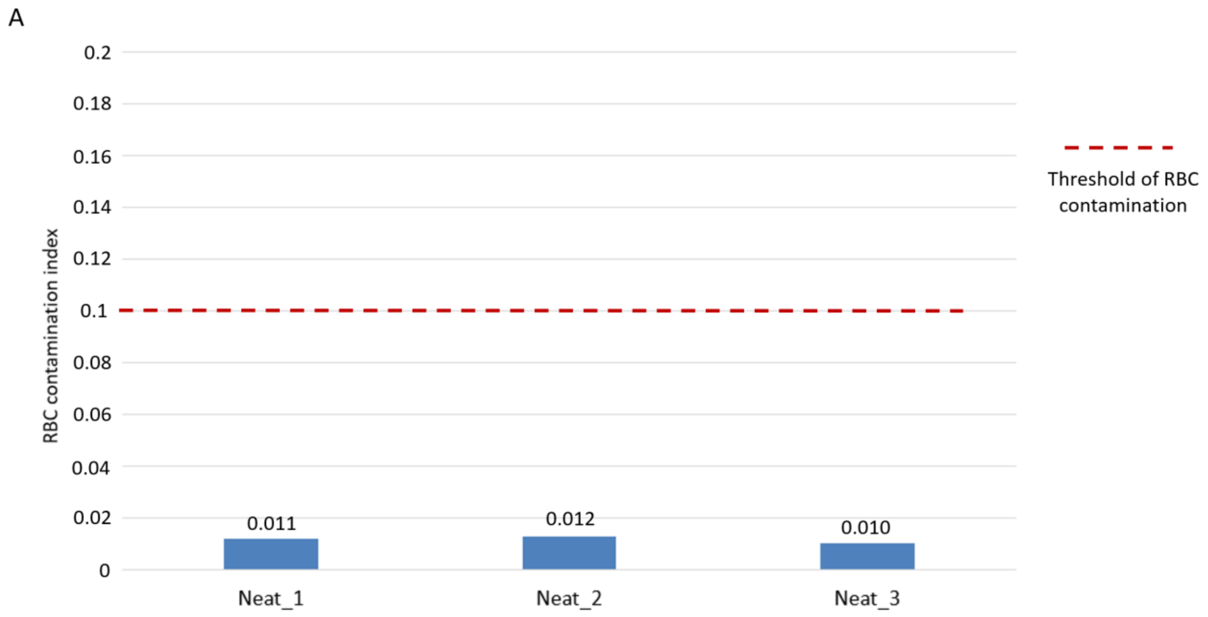

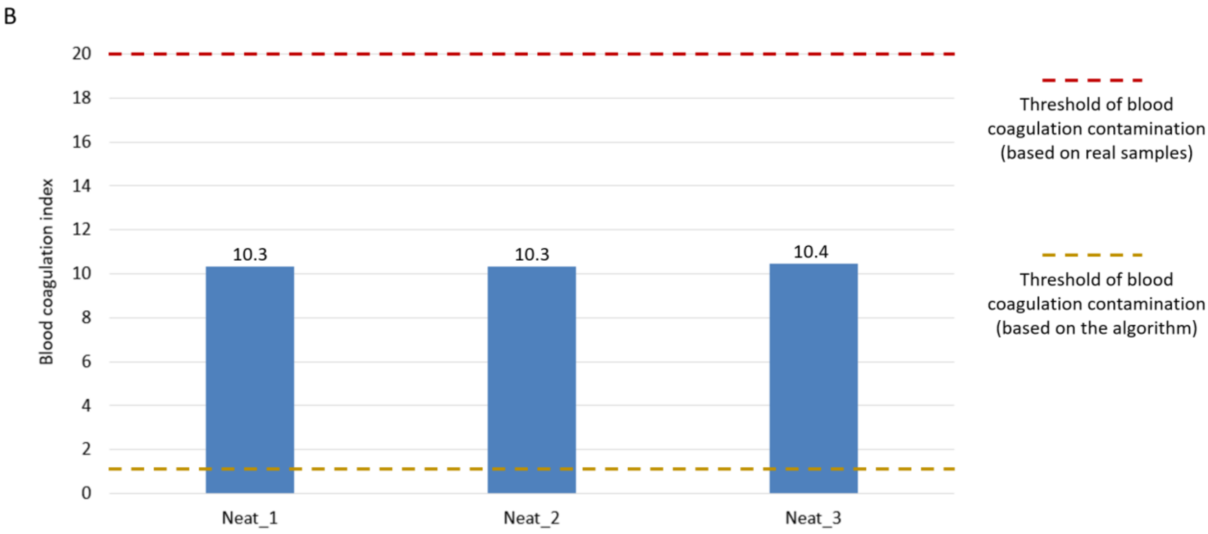

Figure S1. RBC (A) and Coagulation (B) contamination indices based on Baize software.

A

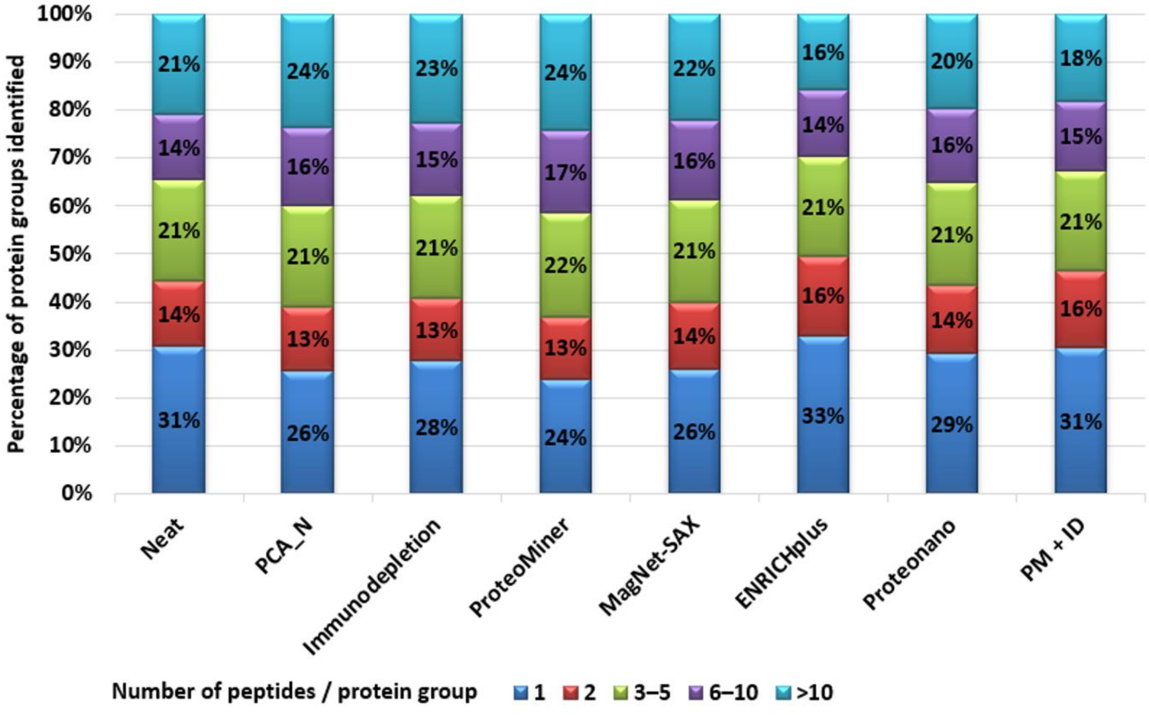

B

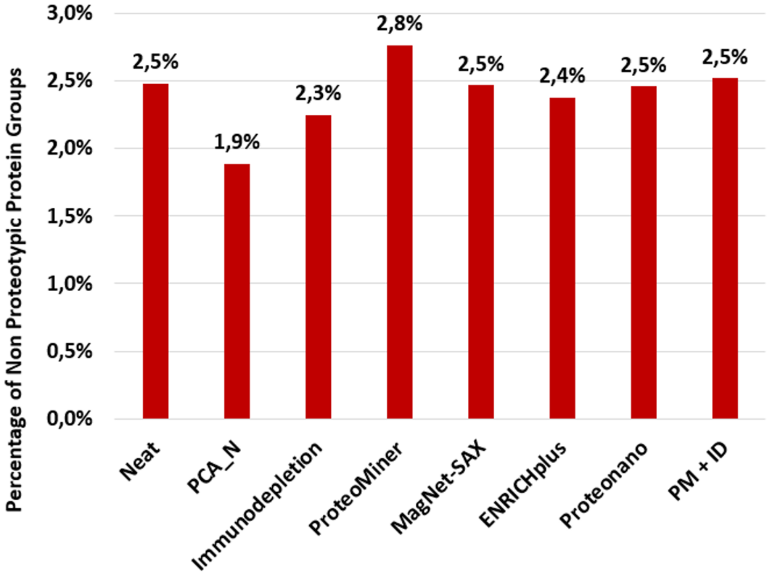

Figure S2. Peptide-to-protein group (A) and non-proteotypic protein groups percentages (B) distribution across workflows.

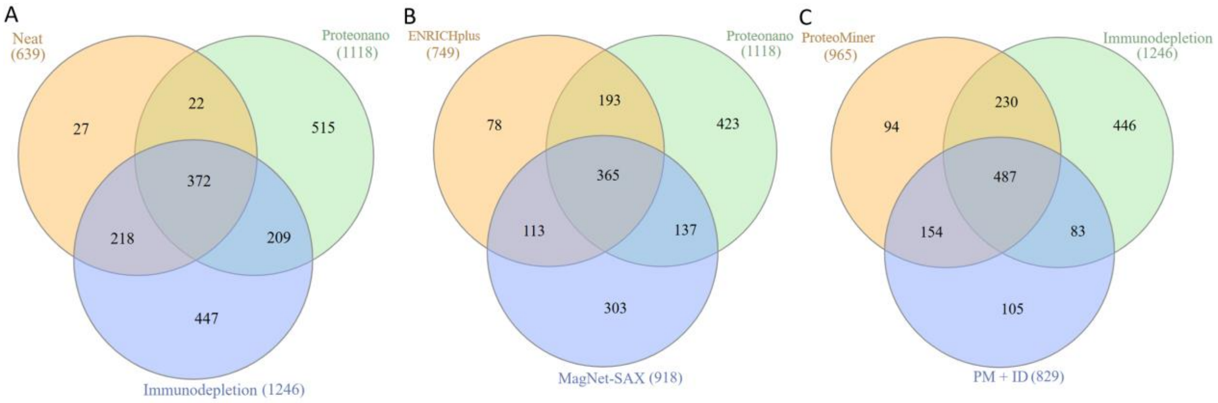

Figure S3. Overlap of confident protein groups identified across workflows.

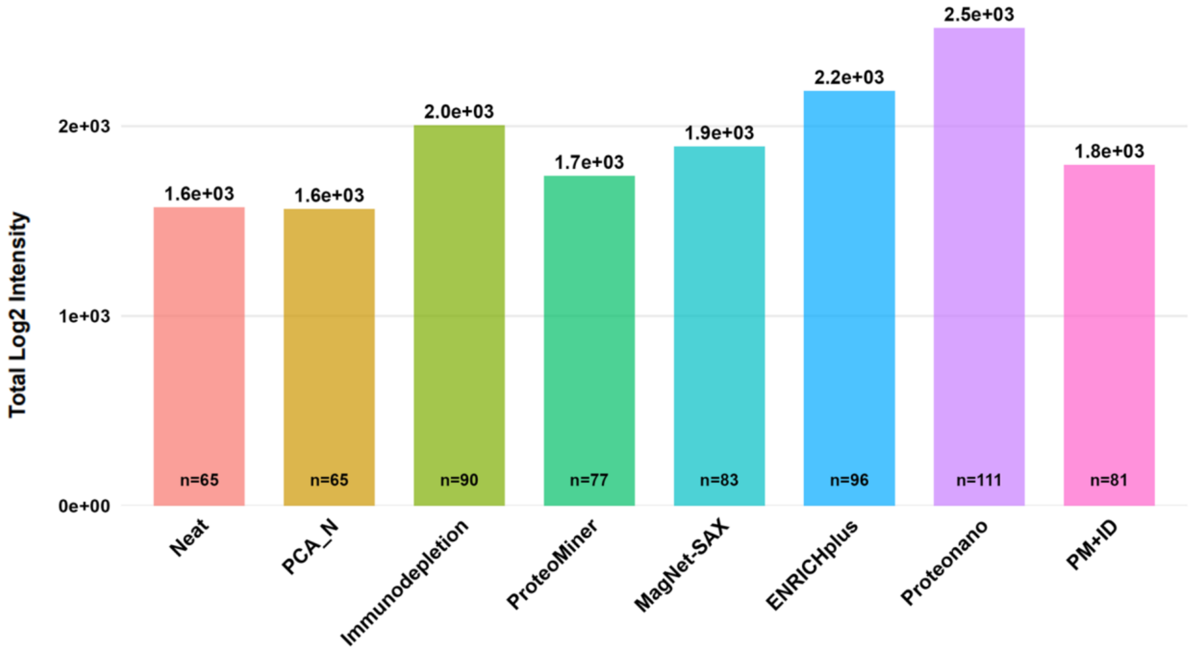

Figure S4. Summed abundances of coagulation proteins across workflows.

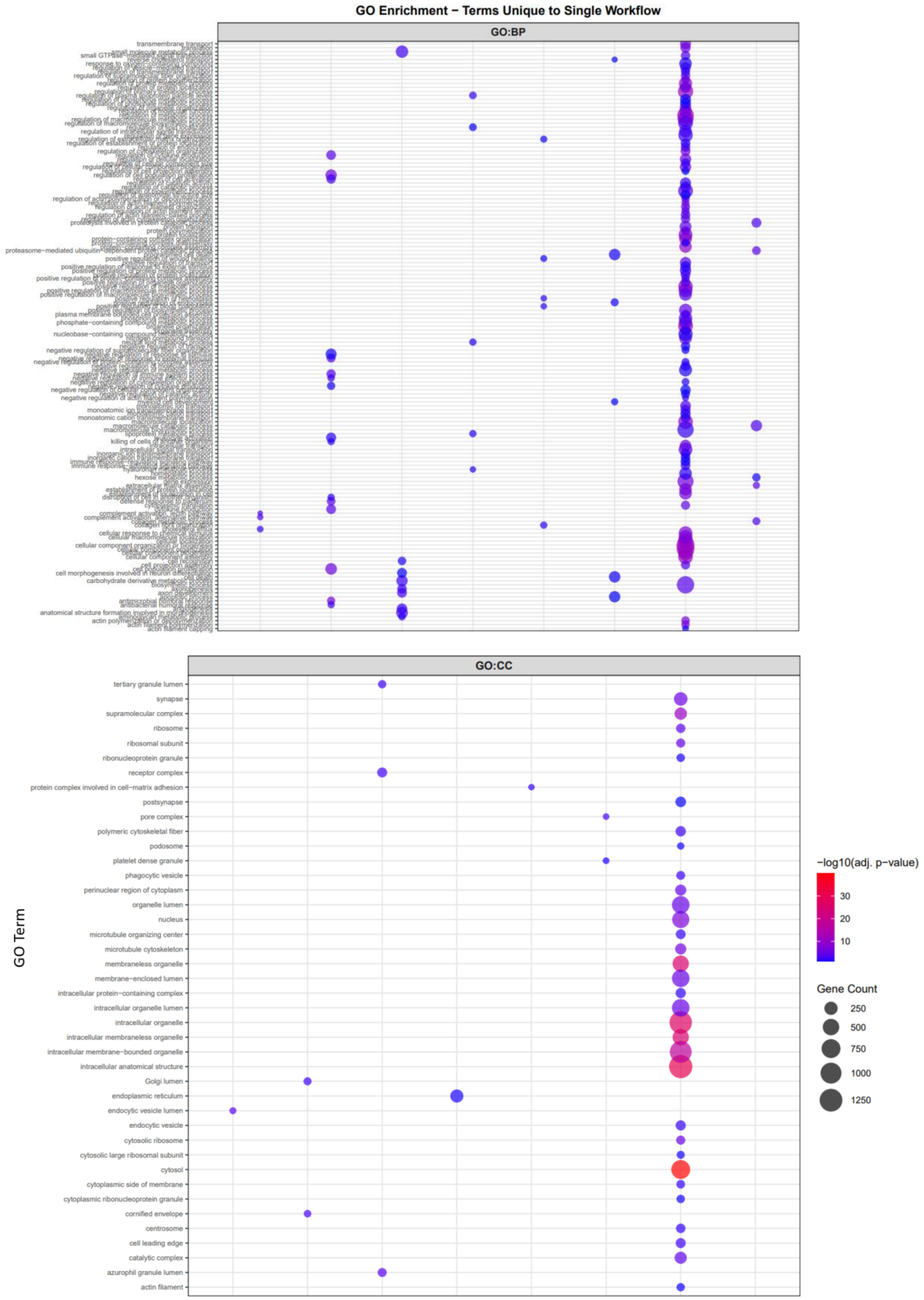

GO Enrichment – Terms Unique to Single Workflow
GO:BP
transmembrane transport
actin filament capping
GO:CC
tertiary granule lumen
synapse
supramolecular complex
ribosome
ribosomal subunit
ribonucleoprotein granule
receptor complex
protein complex involved in cell−matrix adhesion
postsynapse
pore complex
polymeric cytoskeletal fiber
podosome
platelet dense granule
phagocytic vesicle
perinuclear region of cytoplasm
organelle lumen
nucleus
microtubule organizing center
microtubule cytoskeleton
membraneless organelle
membrane−enclosed lumen
intracellular protein−containing complex
intracellular organelle lumen
intracellular organelle
intracellular membraneless organelle
intracellular membrane−bounded organelle
intracellular anatomical structure
Golgi lumen
endoplasmic reticulum
endocytic vesicle lumen
endocytic vesicle
cytosolic ribosome
cytosolic large ribosomal subunit
cytosol
cytoplasmic side of membrane
cytoplasmic ribonucleoprotein granule
cornified envelope
centrosome
cell leading edge
catalytic complex
azurophil granule lumen
actin filament
GO Term
−log10(adj. p−value)
30
20
10
Gene Count
250
500
750
1000
1250

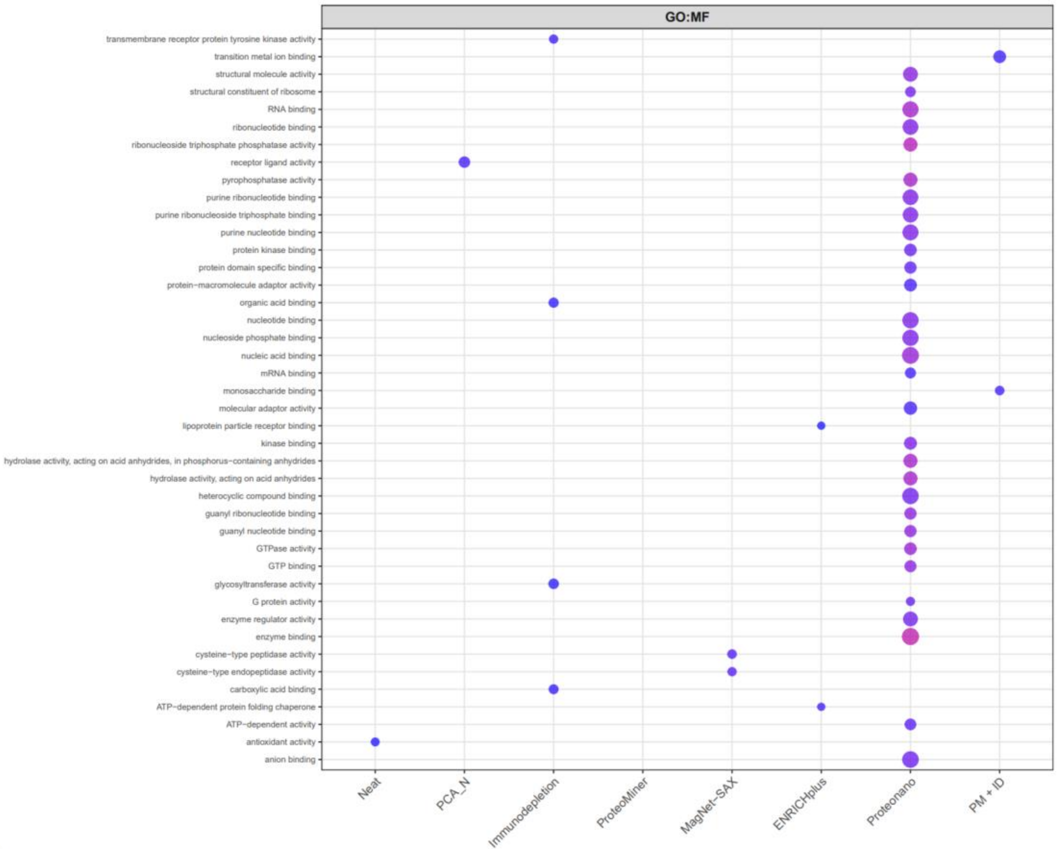

Figure S5. GO term analysis for unique functions across worfklows.

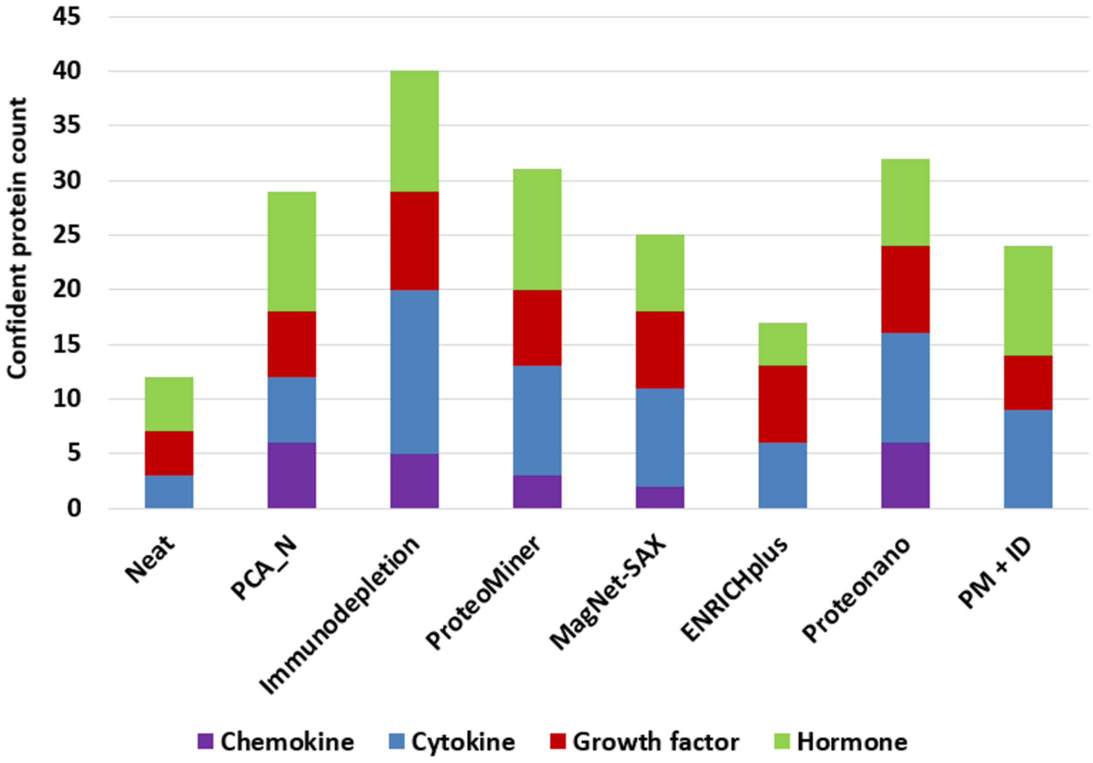

Figure S6. Functional analysis of confident proteins based on the Human protein atlas.

A

B

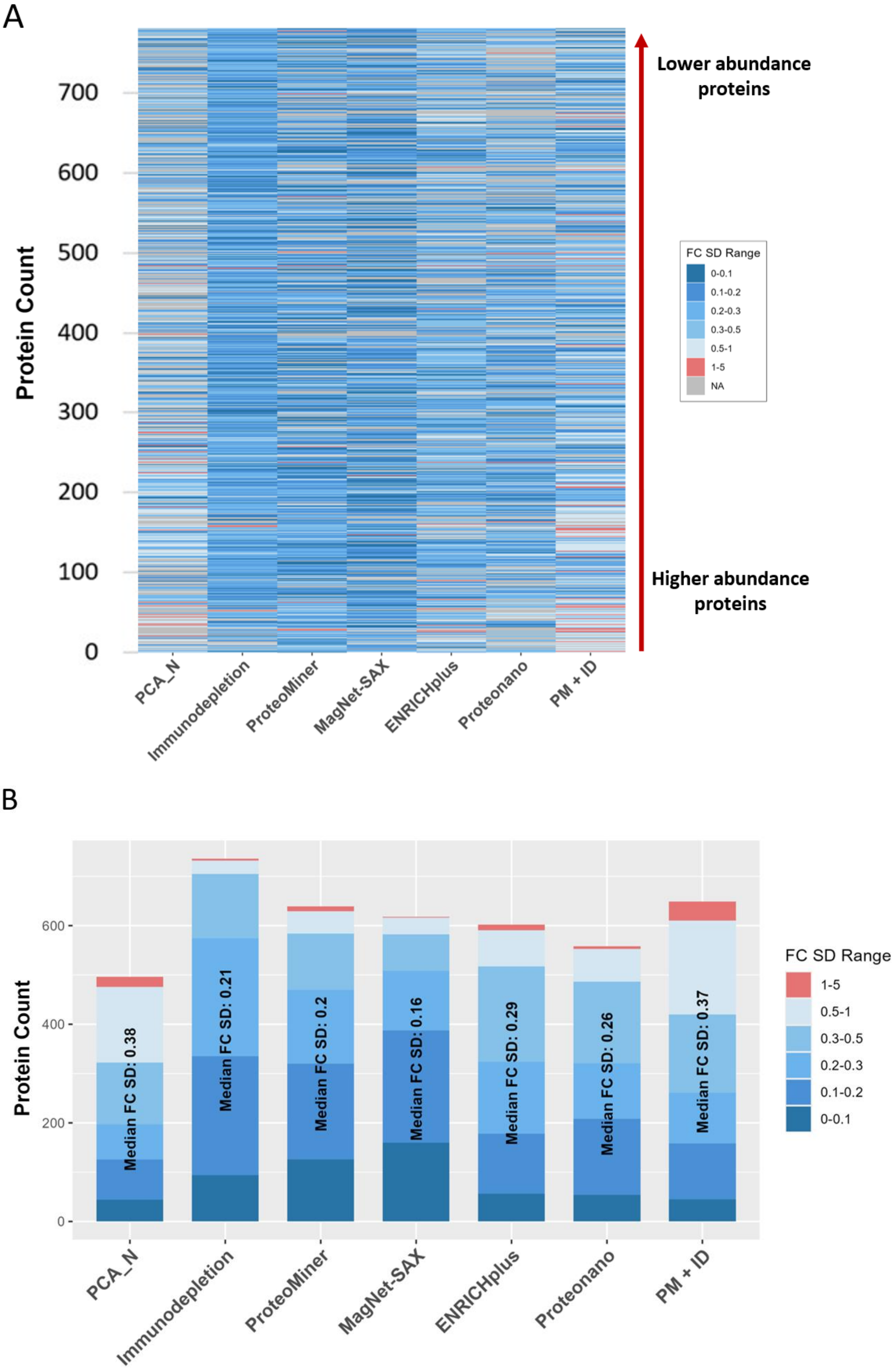

Figure S7. Standard deviation of the fold change values across replicates for each technique (A). Grouping of the standard deviation values from (A) into six ranges: 0–0.1, 0.1–0.2, 0.2–0.3, 0.3–0.5, 0.5–1, and 1–5 (B). The proteins in the panels are ranked in decreasing order of abundance – from bottom to top.

Table S1. Platelet markers quantification in platelet-free plasma.

Platelet marker	Protein.Group	Protein.Names	Genes	First.Protein.Description	Neat_1	Neat_2	Neat_3
CD41	P08514	ITA2B_HUMAN	ITGA2B	Integrin alpha-IIb	0	0	0
CD42a	P14770	GPIX_HUMAN	GP9	Platelet glycoprotein IX	0	0	0
CD61	P05106	ITB3_HUMAN	ITGB3	Integrin beta-3	0	0	0
CD62p	P16109	LYAM3_HUMAN	SELP	P-selectin	205251	146452	0

Table S2. GO term analysis of the uniquely identified proteins across workflows.

Number of exclusive proteins / function	Proteonano	PCA_N	Immunodepletion	PM + ID
Protein binding	273	93	------	------
Catalytic activity	132	------	88	36
Signaling	132	65	------	------

Table S3. Quantitative analysis of the EV markers across various approaches.

EV	Neat	Immunodepletion	PCA_N	ProteoMiner	MagNet-SAX	PM + ID	Proteonano	ENRICHplus
CD9	0	0	0	16,73009	0	0	21,51946	22,43577
TSG101	0	0	0	0	0	0	16,91391	14,00549
FLOT1	0	17,16601	0	16,72644	16,84437	15,93154	22,30685	19,24758
ITGAX	0	0	0	0	0	0	16,99417	0
EZR	12,771758	15,42364	0	11,46347	0	13,98293	16,7211	15,55374
PDCD6IP	14,609941	14,20124	0	13,02721	0	13,6247	17,20293	15,31313
ITGAM	15,972068	15,40728	0	16,18366	0	15,70698	17,44486	16,19876
SDCBP	0	0	0	10,71447	0	14,60692	17,01151	0

Table S4. GO term analysis highlights of specific functions across workflows.

Condition	Function	Adjusted p-value	Number of proteins
Proteonano	G protein activity	0.00033529089737805985	27
PCA_N	Cell adhesion molecules	0.00005056832079923546	47
Immunodepletion	Cell adhesion molecules	0.0004647088348274078	61
